# Molecular Features of the Measles Virus Viral Fusion Complex That Favor Infection and Spread in the Brain

**DOI:** 10.1128/mBio.00799-21

**Published:** 2021-06-01

**Authors:** Cyrille Mathieu, Francesca T. Bovier, Marion Ferren, Nicole A. P. Lieberman, Camilla Predella, Alexandre Lalande, Vikas Peddu, Michelle J. Lin, Amin Addetia, Achchhe Patel, Victor Outlaw, Barbara Corneo, N. Valerio Dorrello, Thomas Briese, Diana Hardie, Branka Horvat, Anne Moscona, Alexander L. Greninger, Matteo Porotto

**Affiliations:** a Center for Host-Pathogen Interaction, Columbia University Vagelos College of Physicians and Surgeons, New York, New York, USA; b Department of Pediatrics, Columbia University Vagelos College of Physicians and Surgeons, New York, New York, USA; c CIRI, Centre International de Recherche en Infectiologie, Team Immuno-Biology of Viral Infections, University of Lyon, Inserm, U1111, Université Claude Bernard Lyon 1, CNRS, UMR5308, ENS de Lyon, Lyon, France; d Department of Experimental Medicine, University of Study of Campania “Luigi Vanvitelli”, Naples, Italy; e Department of Laboratory Medicine, University of Washington Medical Center, Seattle, Washington, USA; f Stem Cell Core Facility, Columbia University Vagelos College of Physicians and Surgeons, New York, New York, USA; g Department of Chemistry, University of Wisconsin–Madison, Madison, Wisconsin, USA; h Center for Infection and Immunity, Mailman School of Public Health, Columbia University, New York, New York, USA; i Department of Epidemiology, Mailman School of Public Health, Columbia University, New York, New York, USA; j Division of Medical Virology, Department of Clinical Laboratory Sciences, University of Cape Town and National Health Laboratory Service, Cape Town, South Africa; k Department of Microbiology, Columbia University Vagelos College of Physicians and Surgeons, New York, New York, USA; l Department of Physiology and Cellular Biophysics, Columbia University Vagelos College of Physicians and Surgeons, New York, New York, USA; University of Pittsburgh School of Medicine; Washington University School of Medicine

**Keywords:** viral fusion, central nervous system infection, host-pathogen interaction, viral evolution

## Abstract

Measles virus (MeV) bearing a single amino acid change in the fusion protein (F)—L454W—was isolated from two patients who died of MeV central nervous system (CNS) infection. This mutation in F confers an advantage over wild-type virus in the CNS, contributing to disease in these patients. Using murine *ex vivo* organotypic brain cultures and human induced pluripotent stem cell-derived brain organoids, we show that CNS adaptive mutations in F enhance the spread of virus *ex vivo*. The spread of virus in human brain organoids is blocked by an inhibitory peptide that targets F, confirming that dissemination in the brain tissue is attributable to F. A single mutation in MeV F thus alters the fusion complex to render MeV more neuropathogenic.

## INTRODUCTION

Despite the availability of an effective measles virus (MeV) vaccine, MeV has not been eradicated and has caused 100,000 to 140,000 deaths globally every year since 2010 (1–3). MeV eradication by vaccination is hindered in part by low vaccination coverage, in part related to hesitancy about vaccine safety (4). Since the current vaccine is a live attenuated viral vaccine, it cannot be used in most immunocompromised people. Measles has been undergoing a global resurgence which may worsen since the SARS-CoV-2 pandemic has led to reduced routine childhood vaccination coverage and may expose more vulnerable individuals to infection (5).

MeV infection can result in fatal complications days to years after the acute phase of the infection when it infects the central nervous system (CNS) (6–8). In a small percentage of cases, subacute sclerosing panencephalitis (SSPE) develops several years after the initial infection, characterized by persistent infection of the brain associated with hypermutated MeV genomic RNA and viral transcripts and defective viral particle assembly (9–11). Measles inclusion body encephalitis (MIBE) occurs in immunocompromised patients weeks to months after infection with wild-type (WT) viruses and, in rare cases, has followed previous versions of the attenuated MeV vaccine that are no longer used (6, 12, 13). MIBE may be associated with viral fusion complexes that do not require cell surface receptors, referred to as hyperfusogenic MeV fusion complexes (14, 15). To date, the mechanisms governing MeV infection and spread in the CNS remain poorly understood, although CNS invasion seems to require the viral fusion (F) protein and may be inhibited by fusion inhibitors (16–19).

MeV initially infects activated CD150(SLAM)-expressing immune cells in the respiratory tract, gaining access to the immune system (20). After reaching the draining lymph nodes, MeV proliferates in CD150-expressing lymphocytes and proceeds to cause viremia. Late in infection, MeV infects respiratory epithelial cells via nectin 4 expressed on the basolateral membranes of these cells; from this location MeV exits the host’s respiratory tract and may be transmitted (21, 22). Infection of a cell by MeV starts with attachment to cell surface receptors, and entry is then mediated by the concerted actions of the MeV receptor binding protein hemagglutinin (H) and F that form a protein complex on the surface of the virus (23, 24). Infected cells synthesize F as a precursor (F0) that is cleaved within the cell to yield the prefusion F complex, comprising three C-terminal F1 subunits that are associated via disulfide bonds with three N-terminal F2 subunits. On the surface of viral particles, this trimeric F structure is kinetically trapped in a metastable conformation (16). F is primed for fusion activation when H glycoprotein engages a target cell entry receptor (i.e., CD150 or nectin 4 for wild-type strains) (20–22). After receptor engagement, H triggers the prefusion F protein to undergo a structural transition, extending to insert its hydrophobic fusion peptide into the host cell membrane. F then refolds into a stable postfusion 6-helix bundle structure, which brings the viral and target cell membranes together to initiate formation of the fusion pore. This refolding is based on the interaction between two complementary heptad repeat (HR) regions located at the N- and C termini of the protein (HRN and HRC). This refolding step can be inhibited by peptides corresponding to these HR regions (25).

MeV bearing F proteins with mutations in the HRC domain have been isolated during CNS infection in several patients (14, 15, 26, 27). A viral sequence recovered from two patients who died from MIBE contained F with the mutation L454W (14), which conferred thermal lability to the metastable F. We have previously shown that this F mutation affects entry into target cells. Recombinant MeV (IC323 strain) expressing green fluorescent protein and bearing the L454W F (MeV-IC323-EGFP-F L454W) spreads in cells that lack a known MeV receptor. In cell-to-cell fusion assays, F bearing the L454W mutation alone mediates fusion independently of the H protein (15). In contrast, other hyperfusogenic viruses are dependent on the H protein for membrane fusion (28). The L454W mutation in F of the MIBE patients could have arisen *de novo* in the CNS (14) or could have been present in the WT viral population and undergone positive selection in the CNS. The origin of this virus could not be determined. One report showed that a virus bearing L454W F can emerge under the selective pressure of certain fusion inhibitors (29), indicating that viruses bearing this neuropathogenic F protein can be found outside the CNS. In recent work, we showed that a virus bearing the L454W F outcompetes WT MeV in the lungs of cotton rats, is 10-fold more lethal in a suckling hCD150 transgenic mouse model of MeV CNS infection, and infects the mouse CNS faster than WT MeV (30).

In an effort to explain the CNS impact of the alteration in MeV F, we studied MeV bearing CNS-adapted fusion complexes in two *ex vivo* models of CNS infection—murine organotypic brain cultures (cerebellum slices from suckling mice) and human brain organoids (fetal 8- to 13-week stage of development). The hyperfusogenic variants from cases of encephalitis spread more efficiently than WT MeV in these models. The infection does not require any known measles receptor, and the extent of infection is inversely correlated with the stability of the MeV F (WT or mutant) in its prefusion state. Spread of virus is blocked by fusion inhibitors that inhibit the refolding of F. An innate immune response is induced in both models, but this response does not block viral spread. Viral evolution in these CNS models led—by distinct routes—toward similarly labile and receptor-independent F proteins, highlighting the critical nature of these features in regulating CNS infection.

## RESULTS

### Measles virus bearing the F glycoprotein bearing L454W is not stable in cell culture.

We and others have previously described mutations in the MeV F glycoprotein (L454W, T461I, and N462K) associated with neuropathogenic measles strains that were either isolated from patients or generated in laboratory settings (14, 15, 27, 30). These mutations made the prefusion state of the F protein less stable. To explore the molecular determinants for altered stability, these mutations were mapped onto structures of the prefusion conformation of MeV F (PDB 5YXW; [16]). The three mutations (L454W, T461I, and N462K) are all located within the HRC domain. Based on the prefusion structure, the side chain of L454 binds within a cleft comprising T314 within the same protomer and L457 within an adjacent protomer (Fig. S1A). The larger tryptophan (W) side chain in the L454W mutant could cause steric hindrance with disruption of this interaction. The T461I and N462K mutations occur in a well-ordered α-helical region of the HRC domain (Fig. S1B). The side chain of T461 resides within a cleft between L354 and L355, and T461I would likely lead to steric clashes between these residues. The side chain of N462 hydrogen bonds with the side chain of S352 in an adjacent helix, and N462K would likely disrupt this H-bond interaction. These three mutations occur in the portion of the HRC domain where the head and stalk regions of the prefusion conformation meet. Interactions at this junction are likely to be important for stabilizing the prefusion state, and consequently, mutations in this region could lead to decreased stability of the MeV-F prefusion structure (15).

10.1128/mBio.00799-21.1 FIG S1 Potential interactions of wild-type (left) and mutated (right) MeV F. (A) L454W and E455G; (B) T461I and N462K; (C) M337L. Mutated residues are shown in magenta. Download FIG S1, PDF file, 2.9 MB. Copyright © 2021 Mathieu et al. 2021 Mathieu et al. https://creativecommons.org/licenses/by/4.0/ This content is distributed under the terms of the Creative Commons Attribution 4.0 International license.

**FIG 1:**
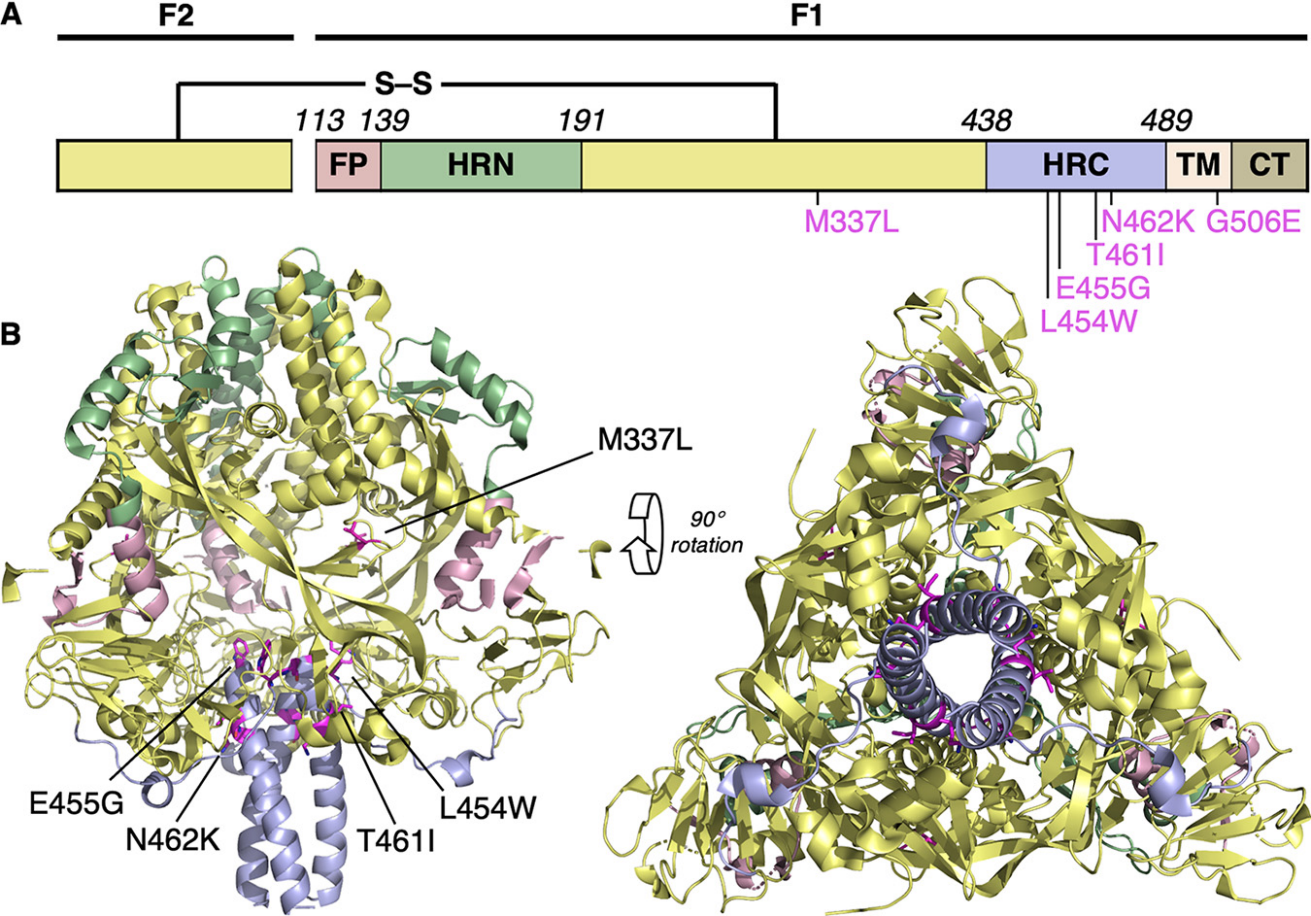
Location of substitutions within the F protein from CNS-adapted virus. (A) Schematic of MeV F with fusion peptide (FP), N-terminal heptad repeat (HRN), C-terminal heptad repeat (HRC), transmembrane (TM), and cytoplasmic (CT) domains indicated. (B) Ribbon diagrams of the prefusion MeV F protein (MeV F; PDB 5YXW [16]). Five substitutions (M337L, L454W, E455G, T461I, and N462K) in the F protein structure are shown.

The MeV F L454W was found in viruses from two separate clinical cases. The mutation decreases the stability of the fusion protein, producing a hyperfusogenic phenotype that allows MeV to spread in Vero cells even in the absence of known receptors. In order to assess the impact of the mutation on viral fitness, we generated a recombinant infectious clone of MeV IC323-EGFP bearing F L454W and grew the recombinant virus in Vero-CD150 cells at either 37°C or 32°C (the lower temperature stabilizes the F protein in its prefusion state [15]). In the course of this process of generating recombinant viruses bearing the L454W F by reverse genetics, a process expected to be routine, an unexpected set of mutations emerged. For both viral preparations, titers remained low, reaching only 2 × 10^5^ PFU/ml at 32°C and ∼5 × 10^5^ PFU/ml at 37°C, compared to WT virus stocks, which generally grow to over 5.10^6^ PFU/ml. We previously noted that the mutant grows to lower titers than WT virus at 37°C (30), suggesting that the F protein harboring the L454W mutation is detrimental for viral growth in cell culture. Next-generation sequencing of these two viral stocks rescued from the same initial plasmid but grown at different temperatures for 3 passages revealed two different viral quasipsecies (31, 32); in addition to the L454W that was present in 100% of the sequences, a G506E mutation emerged at 32°C with an allele frequency of ∼36%, and an E455G mutation emerged at 37°C with an allele frequency of ∼22% (see Table 1). The E455G mutation can be observed in Fig. 1, but G506 is not resolved in the available crystal structures. The G506 resides toward the end of the transmembrane (TM) domain, and mutation to a charged residue within this membrane-embedded segment is very unusual. We are pursuing further studies to determine the structural and functional impact of this finding. We hypothesized that the instability of the L454W F variant favors the emergence of these two new mutations in cell culture. These unexpected mutations provided remarkable investigative tools as discussed below.

**TABLE 1:** Allele frequencies of the indicated viral quasispecies in OBC and in human brain organoids infected with the indicated viruses

Allele frequencies (%) for:											
OBC at 7 dpi for:					Brain organoids at 10 dpi for:				Brain organoids at 20 dpi for:		
Input virus	Sample CM005	Sample CM006	Sample CM007	Sample CM008	Input virus	FA10	FA11				
66% L454W, 34% L454W/G506E	3% L454W, 97% L454W/G506E	11% L454W, 89% L454W/G506E	4% L454W, 96% L454W/G506E	22% L454W, 78% L454W/G506E	62% L454W, 38% L454W/G506E	4% L454W, 96% L454W/G506E	23% L454W, 77% L454W/G506E				
	Sample CM017					FA11	FA11 + HRC4	FA11 + 3g	FA11	FA11	FA11
78% L454W, 22% L454W/E455G	96% L454W, 4% L454W/E455G				78% L454W, 22% L454W/E455G	100% L454W, 0% L454W/E455G	100% L454W, 0% L454W/E455G	98% L454W, 2% L454W/E455G	100% L454W, 0% L454W/E455G	76% L454W, 24% L454W/E455G	100% L454W, 0% L454W/E455G
						FA10	FA11		FA11	FA11	FA11
					0% L454W, 100% L454W/E455G	8% L454W, 92% L454W/E455G	0% L454W, 100% L454W/E455G		0.1% L454W, 99.9% L454W/E455G	1.1% L454W, 98.9% L454W/E455G	0.1% L454W, 99.9% L454W/E455G

### Growth of MeV-IC323-EGFP-F L454W versus WT virus in murine organotypic brain cultures (OBC) in the absence of known receptor.

**FIG 2:**
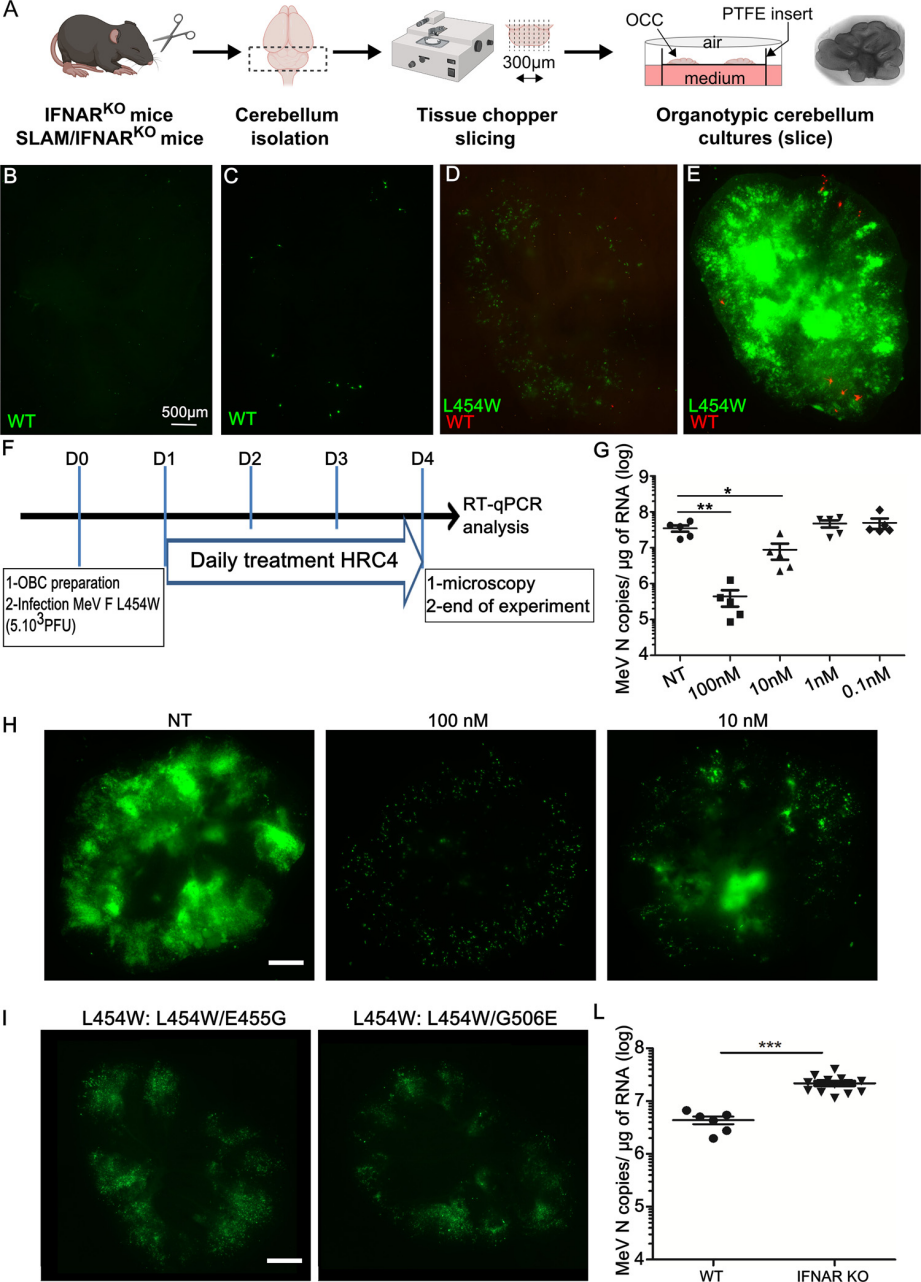
Infection with wild-type (WT) versus virus bearing the L454W F; the CNS-adapted virus outcompetes the WT virus in organotypic brain cultures (OBC). (A) Schematic representation of OBC preparation from the organ, slicing using a tissue chopper II device, and transfer of slices on insert for further culture and infection. (B and C) OBC from IFNAR^KO^ murine brains were infected with 5,000 PFU/slice WT virus bearing EGFP (green fluorescence). (D and E) OBC from IFNAR^KO^ murine brains were coinfected with 5,000 PFU/slice of WT virus bearing tdTomato (red fluorescence) and MeV IC323-EGFP-F L454W (green fluorescence) at 5,000 PFU/slice and monitored over 96 h. Photos were taken at 24 h (D) and 96 h (E). Scale bar = 500 μm. (F to H) MeV F-derived fusion inhibitor peptide (HRC4) inhibits the dissemination of MeV bearing L454W F in OBC. OBC from IFNAR^KO^ murine brains were infected with MeV IC323-EGFP-F L454W at 5,000 PFU/slice for 4 days. OBC were treated at the indicated concentrations or left untreated (NT control) by adding HRC4 fusion inhibitory peptide 24, 48, and 72 h after initial infection. (F) Schematic of the procedure. (G) Total RNA was harvested from organotypic slices at 4 days postinfection (dpi), and the level of MeV N gene expression was quantified by RT-qPCR. Results are expressed as means ± standard deviations of cultures from 5 different mice (**, *P < *0.01; ***, *P < *0.001 [Mann-Whitney-U test]). (H) Green fluorescence related to infection was observed 4 dpi by epifluorescence microscopy in OBC treated at the indicated concentrations (scale bar = 500 μm). (I) *Ex vivo* virus bearing the L454W F infection in fully immunocompetent OBC. OBC from C57BL/6 murine brains were infected with L454W F-bearing virus (using both viral preparations, one with the additional E455G in F and the one with G506E in F) at 1,000 PFU/slice for 7 days. Pictures were taken at 4 days after infection as indicated. (L) L454W-bearing virus growth in WT and IFNAR^KO^ OBC. OBC from WT or IFNAR^KO^ murine brains were infected with 1,000 PFU/slice MeV IC323-EGFP-F L454W for 7 days. Total RNA was harvested from OBC at 4 days postinfection, and the level of MeV N gene expression was quantified by RT-qPCR. Results are expressed as means ± standard deviations in cultures from at least 5 different mice (*, *P < *0.05; ***, *P < *0.001 [Mann-Whitney-U test]). Pictures where reconstituted using the Stitching plugin with ImageJ software.

The viruses bearing F L454W were isolated from the CNS, and we hypothesized that this mutation would be under positive selective pressure in brain models. We previously used mouse cerebellar organotypic brain cultures (OBC) from IFNAR1 knockout (KO) (IFNAR1^KO^) mice to assess viral infection and spread in the absence of a type 1 interferon (IFN) response (33) (see Fig. 2A). OBC from mice that express the human CD150 F1 transgene sustain WT virus infection and spread (34), but the WT virus does not spread in the absence of known MeV receptors (33, 35). In the experiment shown in Fig. 2B to E OBC were derived from IFNAR1^KO^ mice that do not express any known measles receptor. The WT virus (expressing enhanced green fluorescent protein, EGFP) fails to spread over 96 h (Fig. 2B and C). In Fig. 2D and E OBC from IFNAR1^KO^ mice were coinfected with 5,000 PFU of WT virus expressing the red fluorescent protein (tdTomato) and MeV-IC323-EGFP-FL454W (a mixed population containing the two F allele sequences—L454W and L454W/G506E—that emerged in culture). Infection was monitored at 24 h (Fig. 2D) and 96 h (Fig. 2E). While the WT virus did not (as expected) spread well in the OBC, the virus bearing L454W (EGFP) infected and spread, and the G506E mutation allele frequency increased from ∼36% to ∼70%.

### Blocking fusion inhibits spread of all variants.

MeV vaccine strain infection studies have suggested that interfering with F protein function can reduce CNS spread (17). We previously showed that a MeV F-derived dimeric cholesterol-conjugated fusion inhibitory peptide (“HRC4”) blocks infection with WT MeV *in vitro*, *ex vivo*, and *in vivo* in cotton rats and mice (30, 34, 36). The HRC4 peptide’s effect on virus dissemination in IFNAR1^KO^ OBC (no known MeV receptor [33]) after exposure to virus was assessed in Fig. 2F to H. The amount of MeV N RNA copies—reflecting the viral load present in the OBC at the end of the experiment—was quantified using reverse transcriptase quantitative PCR (RT-qPCR). Figure 2G shows the significant 2-log reduction in viral load in the cultures treated postinfection with 100 nM HRC4 peptide (**, *P* = 0.008, Mann-Whitney U-test) and a 4-fold significant reduction in the number of MeV N RNA copies in the group treated with 10 nM peptide (*, *P* = 0.03, Mann-Whitney U-test), compared to the viral load in untreated samples. MeV IC323-EGFP-F L454W virus invaded the OBC, forming extensive areas of fusion throughout the culture 4 days after infection (Fig. 2H). The HRC4 peptide (100 nM or 10 nM) blocks the spread of MeV-IC323-EGFP-F L454W over the same time period (Fig. 2H). At the highest concentration used (100 nM), only isolated single infected cells were observed. The lower concentration (10 nM) was partially inhibitory, with a few focal areas of dissemination (Fig. 2H). The peptide was ineffective at concentrations below 10 nM (Fig. 2G and H). Since the fusion inhibitor was added 24 h after infection, these results indicate that the HRC4 peptides block cell-to-cell spread in three-dimensional CNS-derived OBC, suggesting that MeV dissemination in brain depends on MeV F protein. Even when using OBC from IFNAR1^KO^ mice that express the human CD150 (SLAM) receptor, peptide (100 nM) blocked dissemination of both MeV IC323-EGFP-F L454W and WT (Fig. S2A).

10.1128/mBio.00799-21.2 FIG S2 (A) Inhibition of spread of the L454W F-bearing virus in highly susceptible CD150xIFNAR^KO^ CNS. MeV HRC4 peptide inhibits dissemination of MeV bearing L454W F in organotypic brain cultures (OBC). Cerebellar slices from CD150xIFNAR^KO^ murine brains were infected with 5,000 PFU/slice of MeV IC323-EGFP-F-L454W for 4 days. OBC were treated at the indicated concentrations by adding HRC4 fusion inhibitory peptides 24 h after initial infection or untreated (i.e., NT, not treated-control received DMSO). Total RNA was harvested from organotypic slices at 4 days postinfection, and the level of MeV N gene expression was quantified by RT-qPCR. Results are expressed as means ± SD in cultures from at least 5 different mice (*, *P < *0.05; ***, *P < *0.001 [Mann-Whitney U-test]). (B) Infection of human brain organoids in the presence of fusion inhibitors. FA11 iPSC-derived brain organoids were infected with MeV IC323-EGFP-F-L454W viruses (3 separate wells containing 2 to 4 organoids were infected with each virus). Then, 3 h after infection, the viral inoculum was aspirated and medium was added, and 24 h after infection, 1μM of fusion inhibitor dimeric lipopeptide (HRC4) or 10 μM of 3G was added to the brain organoids. Images were taken after 10 days, before lysis. Bar = 100 μm (C) Viral genome copies calculated from qPCR analysis of RNA extracted from brain organoid lysate. Download FIG S2, TIF file, 2.4 MB. Copyright © 2021 Mathieu et al. 2021 Mathieu et al. https://creativecommons.org/licenses/by/4.0/ This content is distributed under the terms of the Creative Commons Attribution 4.0 International license.

### Evolution of the L454W-bearing viruses in immunocompetent OBC.

To explore the determinants of MeV fitness in the CNS, we evaluated the evolution of the two viruses bearing a double population of L454W F plus either L454W/E455G F or L454W/G506E F. RNA virus populations, including MeV, are present as quasispecies which can undergo rapid adaptation to their growth environment (31, 32). The MeV L454W F stocks represent viral quasispecies in which the E455G and G506E variants are major components. To assess the evolution of these viruses under the selective pressure of growth in fully immunocompetent mouse CNS models, we derived OBC from WT mice. OBC from C57BL/6 suckling mice were infected with MeV WT and the set of L454W F viruses. We previously observed that WT virus did not spread in this model (35), and this finding was confirmed here, but in contrast, both viruses bearing the L454W F spread in the C57BL/6 OBC with similar levels of infection after 7 days (Fig. 2I). Infected OBC were lysed at 7 days for sequencing to assess the transcriptome, quantify viral RNA, and evaluate viral evolution during infection. In C57BL/6 OBC, viruses bearing the L454W F induced a gene expression pattern associated with intense interferon signaling compared to the OBC infected with WT virus (with minimal spread), where the gene pattern was indistinguishable from that of uninfected OBC (see Fig. S3 for immune staining with specific neuronal markers and Fig. S4A for differential gene expression analysis). Despite this strong innate immune response, the spread of L454W F bearing-virus was not halted (see difference between viral genome in WT versus IFNAR^KO^ murine OBC in Fig. 2L). Viral sequence analysis is presented in Table 1 and in the LAVA plots in Data Sets S1 and S2 in the supplemental material (37). Table 1 and the murine OBC LAVA plot in the supplemental material show the allele frequency for L454W/G506E in four samples (CM005-8) and for L454W/E455G in one sample (CM017). The L454W/G506E input virus had an allele frequency for the double mutant of ∼36% that rose after 7 days in OBC to 97%, 89%, 96%, and 78%. For the L454W/E455G, however, the input virus had an allele frequency for the double mutant of ∼22%, and after *ex vivo* growth the double mutant decreased to ∼4%.

10.1128/mBio.00799-21.3 FIG S3 Cell population present in organotypic brain cultures (OBC) the day of infection. Briefly, OBC from SLAM mice were fixed and embedded in paraffin. (A to D) Slices of 10 μm thickness were stained in red for (A) granular neurons (NeuN) and Purkinje/Golgi neuronal cells (CB28K), (B) astrocytes (GFAP), (C) microglia (IBA1), and (D) oligodendrocyte (OliG2 and MBP). Slices were counterstained with DAPI (in blue). Scale bar = 100 μm. Download FIG S3, PDF file, 0.2 MB. Copyright © 2021 Mathieu et al. 2021 Mathieu et al. https://creativecommons.org/licenses/by/4.0/ This content is distributed under the terms of the Creative Commons Attribution 4.0 International license.

10.1128/mBio.00799-21.4 FIG S4 (A) Induction of interferon-stimulated genes by MeV IC323-EGFP-F-L454W compared to wild-type MeV in mouse brain slice cultures. Differential expression analysis was performed on mouse brain slices that were either uninfected (*n* = 2), WT-infected (*n* = 3), or infected with L454W F-bearing virus (*n* = 5). RPM values for MeV for each sample are depicted below each heatmap. Raw counts were normalized across all samples, and differential expression analysis was performed. The 50 genes with the lowest adjusted *P* value when comparing L454W F-bearing virus infection with uninfected organoids are depicted in the heatmap, colored by log2 fold change of each sample relative to the mean normalized counts for each gene. (B) RPM values for MeV for each sample are depicted below each heatmap. Coloring is by log2 fold change of each sample relative to the mean normalized counts for each gene. (C) Induction of host antiviral genes in human brain organoids infected with fusion protein mutant MeVs. The expression of the 50 genes with the lowest adjusted *P* value when comparing L454W F-bearing virus infection with uninfected organoids was examined across 5 MeV viruses (WT-eGFP, WT-TdTomato, F-L454W, F-L454W/E455G, F-T461I, and N462K; *n* = 2 each; each point is derived from triplicate wells with 2 to 4 brain organoids in each well). (D) RPM values for MeV for each sample are depicted below each heatmap. Coloring is by log2 fold change of each sample relative to the mean normalized counts for each gene. Download FIG S4, PDF file, 0.2 MB. Copyright © 2021 Mathieu et al. 2021 Mathieu et al. https://creativecommons.org/licenses/by/4.0/ This content is distributed under the terms of the Creative Commons Attribution 4.0 International license.

### Infection of human induced pluripotent stem cell (hiPSC)-derived brain organoids—CNS adapted variants versus WT virus.

**FIG 3:**
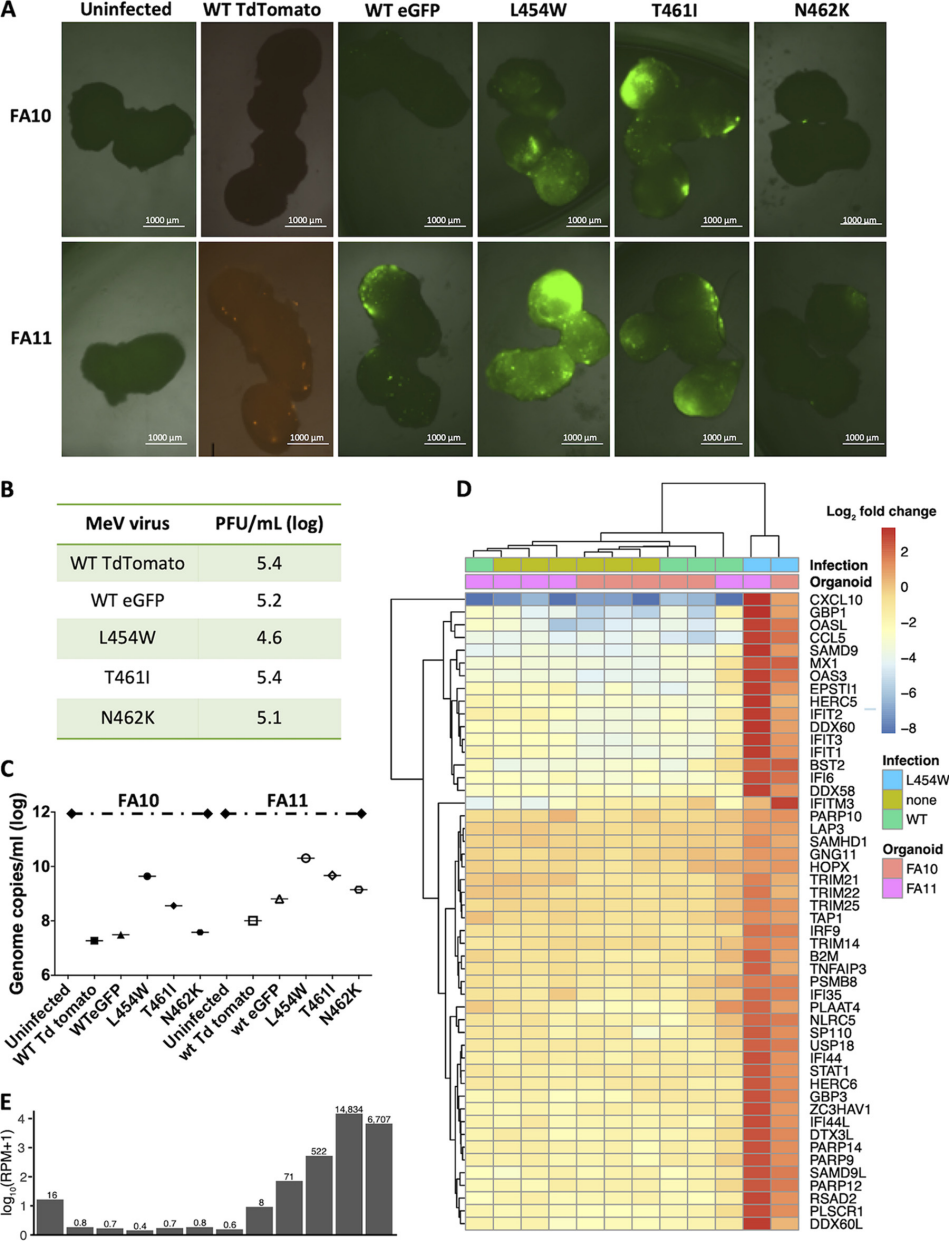
CNS-adapted MeV variants spread efficiently in human pluripotent stem cell (hiPSC)-derived brain organoids. (A) Two separate sets of 90-day-old human brain organoids (derived from two hiPSCs, FA10 and FA11) were infected with recombinant MeV viruses (with either EGFP or tdTomato fluorescent protein) bearing the indicated MeV fusion (F) proteins. For each virus, 3 separate wells each containing 2 to 4 organoids were infected (5,000 PFU/well). The brain organoids were monitored over time, and the fluorescence shown here reflects the infection after 10 days. Bar = 1,000 μm. (B) Viral titer of the inoculum used for infection was assessed on Vero CD150 cells (PFU/ml, log). (C) Total RNA was harvested from the human brain organoids at 10 days postinfection, and the level of MeV N gene expression was quantified by RT-qPCR. (D) RNA-Seq analysis of WT versus L454W F-bearing virus infection in brain organoids (data from three separate experiments). Seven replicates of uninfected and MeV-infected brain organoids were transcriptionally profiled (*n* = 6 uninfected, *n* = 5 WT, *n* = 2 L454W F-bearing virus). (E) RPM values for MeV for each sample are depicted in the same order as for the heatmap in panel D. Raw counts were normalized across all samples, and differential expression analysis was performed. The 50 genes with the lowest adjusted *P* value between MeV IC323-EGFP-F L454W and uninfected are depicted in the heatmap, colored by log_2_ fold change of each sample relative to the mean normalized counts for each gene.

To validate the pattern of immune gene response and explore the surprising finding that the L454W/G506E virus—originally identified as a laboratory adaptation—underwent positive selection in mouse OBC, we used human brain organoids. For this human tissue model, we differentiated hiPSC from two separate donors—one male (FA10) and one female (FA11)—into brain organoids (38). Ninety-day old organoids were infected with viruses bearing WT or L454W F and also for comparison with MeV bearing N462K F (a lab adaptive mutation previously found to grow well in hamster brains) and T461I F (from an SSPE case) (27) (see Fig. 1). Infection and spread of fluorescent virus were monitored over a 10-day period (Fig. 3A and Fig. S1B show photos at day 10). To ensure that equal amounts of virus were used, the inocula used in the human brain organoids were assessed in parallel in Vero cells expressing CD150 (Vero-CD150) (Fig. 3B). All the viral titers (PFU/ml) were similar, with the MeV L454W F being slightly lower. After 10 days, the human brain organoids were lysed for RNA sequencing to assess the transcriptome, quantify viral RNA, and evaluate viral evolution during infection (Fig. 3C to E, Fig. S4C and D). The viruses bearing L454W F spread more than the virus bearing the WT F (Fig. 3A and C) and were blocked by the HRC4 fusion inhibitor added 24 h after infection (Fig. S2B and C). The MeV T461I F (SSPE patient) also spread, but in contrast, the MeV N462K F (lab adapted) spread only slightly better than WT virus (Fig. 3A and C).

The differential gene expression in uninfected or infected human brain organoids (WT MeV or MeV L454W F) is shown in Fig. 3D. MeV L454W F was present at severalfold higher reads per million (RPM) values than MeV WT (Fig. 3E). MeV L454W F induced a gene expression pattern that was associated with interferon signaling, compared to organoids infected with WT virus and control samples (*P* = 2.6 × 10^−17^, see Fig. S4C and D for the differential gene expression analysis). This difference seems likely related to the differences in viral growth, since it seems to correlate with viral genome count regardless of the specific F mutation. Fig. S4C shows the gene expression in organoids infected with the MeV variants shown in Fig. 3A). For both sets of human brain organoids, the highest gene-level expression coefficient correlated with the youngest fetal development stages (8 to 13 postconception weeks) and with brain tissues derived from the amygdala (Fig. S5A) with specific CNS markers (Fig. S6), as noted previously (39, 40). The developmental stage was not affected by infection. Pathway analysis of differentially expressed genes common to the two human brain organoid infection series confirmed that a strong interferon response was evoked by measles infection in human brain organoids (Fig. S5B).

10.1128/mBio.00799-21.5 FIG S5 (A) Correlation heatmap of gene-level RPKM values between brain organoids and the BrainSpan atlas. Correlation coefficients were determined between two organoids (FA10 and FA11) uninfected or infected with MeV variants and a set of 524 fetal and adult brain transcriptome datasets. The top 100 BrainSpan samples with the highest correlation coefficients to the uninfected FA10 brain organoid (first replicate) are depicted for all brain organoid samples. From three differentiations as indicated. (B) KEGG pathway analysis of genes differentially expressed upon MeV IC323-EGFP-F-L454W infection. Genes with absolute Log2 fold change of  >1 and adjusted *P* value of <0.0001 (*n* = 124) were tested for enrichment in KEGG pathways. The 20 pathways with the lowest *P* value are shown. The number of genes in each pathway is depicted by the size of the dot and colored by adjusted *P* value of the statistical enrichment. Download FIG S5, PDF file, 0.4 MB. Copyright © 2021 Mathieu et al. 2021 Mathieu et al. https://creativecommons.org/licenses/by/4.0/ This content is distributed under the terms of the Creative Commons Attribution 4.0 International license.

10.1128/mBio.00799-21.6 FIG S6 Cell population present in brain organoids. Representative sections of 30-day-old brain organoids showing expression-specific markers for progenitor (nestin, Pax-6), neuronal (NeuN, MAP2), and glial (astrocytes, GFAP; oligodentrocytes, Olig1 and Olig2) cells by immunofluorescence. Scale bar = 100 μm. Download FIG S6, PDF file, 1 MB. Copyright © 2021 Mathieu et al. 2021 Mathieu et al. https://creativecommons.org/licenses/by/4.0/ This content is distributed under the terms of the Creative Commons Attribution 4.0 International license.

Viral sequence analysis from growth in human brain organoids is presented in Table 1 and the LAVA plots (see supplemental material). For the infection with MeV L454W/G506E, the input virus had an allele frequency for the double mutant of ∼38%, and this increased to ∼96% in FA10 and ∼77% in FA11 brain organoids (see Table 1 and human brain organoid LAVA plots in the supplemental material). For the MeV L454W/E455G, the input virus had an allele frequency for the double mutant of ∼22%, and during infection of a second set of brain organoids (derived from FA11 iPSC), the allele frequency of the E455G F decreased to lower than 2% (see Table 1 and human brain organoid LAVA plots in the supplemental material). MeV WT did not show any alteration (see Table 1 and human brain organoid LAVA plots in the supplemental material).

### Additional mutations in the L454W F background stabilize the prefusion state of the F protein.

**FIG 4:**
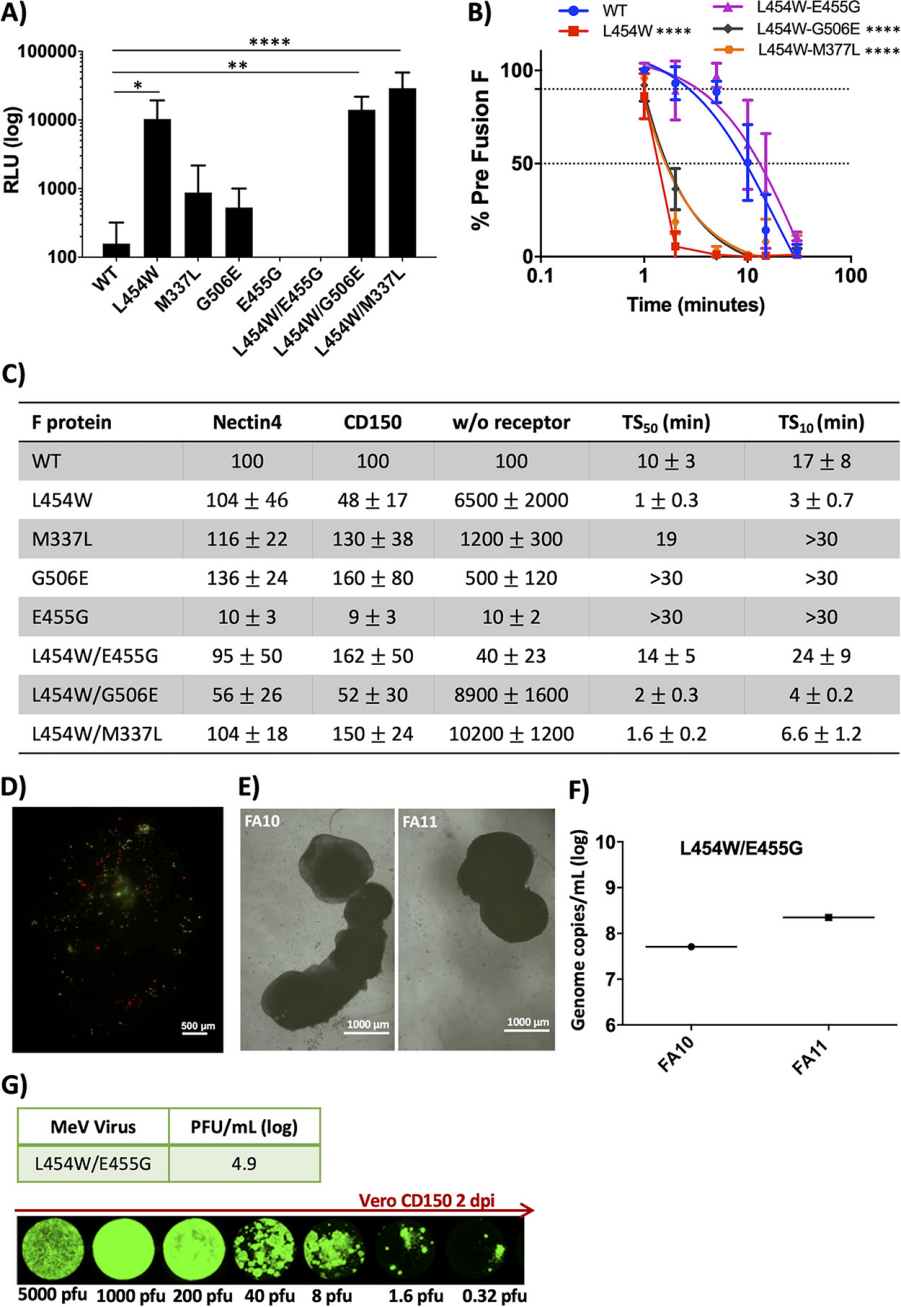
Fusion activity and thermal stability of MeV fusion (F) proteins bearing the indicated mutations. (A) Cell-to-cell fusion between HEK293T cells coexpressing the indicated MeV F proteins + MeV WT hemagglutinin (H) and HEK293T cells (no known measles receptor) was assessed by a B-gal complementation assay. The values on the *y* axis are expressed as relative luminescence unit (RLU) averages (with standard error, SE) of results from three independent experiments. ****, *P* < 0.0001 (2-way analysis of variance [ANOVA]). (B) HEK293T cells were transfected with MeV F protein bearing the indicated mutations and incubated at 37°C for 24 h and then raised to 55°C for the indicated times (*x* axis). The values on the *y* axis represent the percentages of prefusion conformation specific antibody binding to the indicated F proteins (compared to the WT F protein at time zero). The values are the average of three independent experiments. ****, *P* < 0.0001 (2-way ANOVA). (C) Fusion mediated by cells coexpressing MeV H and F proteins bearing the indicated F mutation in the presence of nectin 4, CD150, or no receptor compared to WT F (100%). HEK293T cells were transfected with MeV F protein bearing the indicated mutations and incubated at 37°C for 24 h and then raised to 55°C. The time (minutes) at 55°C that decreases the fraction of prefusion epitope to 50% (TS_50_) and to 10% (TS_10_) is indicated compared to the WT F at time zero (100%). Data are averages from three experiments +/− SE. (D) OBC from IFNAR^KO^ murine brains were coinfected with 5,000 PFU/slice of WT virus bearing tdTomato (red fluorescence) and MeV-IC323-EGFP-F L454W/E455G (green fluorescence) and monitored over 96 h. Photos were taken at 96 h. Pictures where reconstituted using the Stitching plugin with ImageJ software. Scale bar = 500 μm. (E) Two separate sets of 90-day-old human brain organoids (derived from two hiPSCs, FA10 and FA11) were infected with recombinant MeV viruses bearing the L4545W/E455G F. Three separate wells containing 2 to 4 organoids were infected (5 000 PFU/well). The brain organoids were monitored over time, and the fluorescence shown here reflects infection after 10 days. Bar = 1,000 μm. (F) Total RNA was harvested from the human brain organoids at 10 days postinfection, and the level of MeV N gene expression was quantified by RT-qPCR. (G) Viral titer of the viral inoculum used for infection was assessed on Vero CD150 cells (PFU/ml). Photos show the extent of infection after 2 days (PFU/well are indicated). Pictures were reconstituted using the Stitching plugin with ImageJ software.

Since positive selection of G506E F and negative selection of E455G F were observed in both murine OBC and human brain organoids, we compared the functional properties of these F proteins bearing the additional mutations, both alone and in combination with L454W. All the F proteins bearing mutations that were selected in the CNS can mediate cell-to-cell fusion when expressed on transfected cell surfaces, without receptor (Fig. 4A and C). The L454W/G506E F was still significantly less stable than WT F in our functional assays, while the L454W/E455G F was more stable than WT F (Fig. 4B). The hallmark of the neuropathogenic variants is the ability to fuse without a known viral receptor, and the L454W/G506E F can mediate fusion without any of the known receptors (CD150/SLAM or nectin 4). However, the L454W/E455G F, bearing the E455G mutation that dramatically decreased in frequency during growth in brain organoids, does not mediate fusion without a known receptor.

Based on the finding (Fig. 4A to C) that the frequency of E455G decreased during growth in pressure in organoids and that this mutation conferred a receptor dependency on the L454W F, we hypothesized that a homogenous viral population of MeV L454W/E455G F behaves like WT MeV in the organotypic brain slices and in brain organoid models and that E455G would be subject to negative selection pressure in these neural models. We thus attempted to generate recombinant viruses bearing either the L454W/E455G F or the E455G F to analyze their growth and spread in brain organoids. The recombinant virus bearing L454W/E455G F grew similarly to WT virus in Vero-CD150. However, a recombinant virus bearing the singly mutated E455G F could not be recovered, suggesting that it is detrimental in culture. To determine whether the double mutant’s phenotype resembles that of the WT, we coinfected with WT F (red)- and L454W/E455G F (green)-bearing viruses in OBC as shown in Fig. 2D. Figure 4D shows the extent of viral spread 96 h postinfection. In clear contrast to the results shown Fig. 2D (where the MeV L454W F invaded the entire OBC and WT infection was limited) the coinfection resulted in similar (limited) spread for both MeV WT and MeV L454W/E455G F viruses. Infection with fluorescent MeV L454W/E455G F in 90-day-old human brain organoids was monitored for 10 days (Fig. 4E). To ensure that infection was performed with the intended amount of virus (5, 000 PFU/well), the inocula used in the human brain organoids were assessed in parallel in Vero-CD150 cells (Fig. 4G). The MeV L454W/E455G F had only limited spread in both FA10 and FA11 hiPSC-derived brain organoids after 10 days (Fig. 4E) but infected and destroyed Vero-CD150 cell monolayers in 2 days (Fig. 4G). After 10 days, the human brain organoids were lysed for RNA sequencing to assess the transcriptome, quantify viral RNA, and evaluate viral evolution and differential gene expression during infection (Fig. 4F, Fig. S3C and D, Fig. S5A and supplemental material). The amount of viral genome of the infection from MeV L454W/E455G F was similar to what we observed for the WT virus (Fig. 3). After 10 days, the double mutation remained stable in the brain organoids derived from hiPSC FA11, but an ∼8% reversion to E455 in the brain organoids derived from hiPSC FA10 was observed (Table 1 and human brain organoid LAVA plots in the supplemental material). We therefore differentiated another set of brain organoids from hiPSC FA11 and infected them with MeV WT, MeV L454W F (the mixed population of MeV L454W F and MeV L454W/E455G F), and MeV L4545/E455G F, each in triplicate. At 20 days postinfection the brain organoids were lysed, and RNA was extracted for viral sequencing (Table 1). The WT virus underwent only a change in the P gene (R77C) with an allele frequency of ∼30% in all three samples. The results for the MeV L454W F-bearing virus (mixed population of MeV L454W F and MeV L454W/E455G F) were different among the triplicate samples. In two samples, the E455G mutation was totally eliminated. In the third sample, E455G remained at ∼24%. The double mutant MeV L454W/E455G F remained stable with minor changes (the single-population L454W increased 0.1% to 1.1.%).

In light of the surprising finding that an F-stabilizing mutation like G506E (Fig. 4) does not necessarily interfere with CNS adaptation, we searched for evidence of such a phenotype *in vivo*. A clinical sample isolated from the original MIBE cohort that revealed the L454W mutation in F (14) bore an additional mutation in F (M337L). We assessed the effect of M337L on fusion and stability of F, both singly and in combination with L454W (Fig. 4A–C). The expressed M337L F did not mediate fusion in the absence of receptor, and its prefusion state was more stable than that of WT F. The M337L/L454W F promoted fusion in the absence of receptor and was significantly less thermostable than WT F. Evolution *in vivo* led—through a different mutated residue—to a similar functional alteration to that caused by G506E in F. These results reflect a CNS-specific pattern of adaptation.

## DISCUSSION

In a South African MeV outbreak, 8 HIV-infected patients died of MeV CNS manifestations (41). The fusion complexes of MeV isolated from the CNS of patients with MIBE are altered so that F is activated without a known entry receptor (15). The F proteins of the isolates from two separate patients contain one specific amino acid alteration at position 454 (L454W) that increases F’s ability to mediate fusion with any heterotypic attachment protein and markedly decreases F’s thermal stability (15). An unexpectedly informative set of mutations emerged from the process of generating recombinant viruses bearing the L454W F by reverse genetics. The standard methods carried out at 37°C resulted in a mixed population of viruses bearing either L454W F or L454W/E455G F. Lowering the temperature of viral production to 32°C to stabilize the F resulted in a population containing both L454W and L454W/G506E. While E455 is located in the HRC domain of F (see Fig. 1), the residue G506 is in the transmembrane domain and is not resolved in the available crystal structures of MeV F. Placing a charged residue into the transmembrane domain would likely have a drastic impact on protein folding and activity, and yet this mutation was positively selected in our *ex vivo* models. The HRC mutation E455G increases the stability of F, and the L454W/E455G F (in contrast to L454W) requires engagement of H (MeV receptor binding protein) to the receptor in order to mediate fusion. This second HRC domain mutation thus confers a requirement for H-receptor interaction during infection, onto the receptor-independent L454W F. The G506E transmembrane domain mutation slightly restored F’s stability; however, the double mutant L454W/G506 F is still less stable than the WT and mediates fusion in the absence of a known receptor. Although arising as a result of a technical process, these compensatory mutations provided direct evidence that viruses that can fuse without known receptors are positively selected in the CNS.

Fusion-inhibitory peptides inhibit MeV L454W F infection in murine OBC and human brain organoids, in support of the role of F-mediated fusion in CNS spread. Therapies designed to stabilize the prefusion F are in an advanced stage of development for a related virus, respiratory syncytial virus (42). For MeV, in previous work, a small F-targeting molecule (3G) that inhibited WT MeV infection *in vitro* was identified using WT MeV F for screening, and resistance to this inhibitor quickly arose via mutation at position 462 (N462K), one of the mutants we analyzed here (43–47). The mutated F was inherently destabilized but retained in the prefusion state in the presence of the inhibitor 3G (43, 44). The 3G inhibitor did not block spread of MeV L454W F in the human brain organoids (Fig. S2B and C). Another fusion inhibitor, FIP (29), which has a mechanism of action similar to that of 3G (16, 29), has also been shown to exert selective pressure for the emergence of L454W F. We asked whether the selective pressure of FIP would favor MeV L454W F over the double mutant MeV L454W/E455G F in immortalized cells, thereby suggesting that its instability is a disadvantage in culture. We grew viruses bearing L454W and L454W/E455G F in the presence of FIP, and the allele frequency of L454W/E455G decreased from ∼22% to ∼10%. FIP provided a selective pressure that reduced the population of the double mutant. This virus stock bearing L454W F and 10% double mutant was then used to infect Vero cells that bear no known receptor, and the double mutant population rose again, indicating that the lack of receptor is not the primary or only necessary selective pressure to maintain the unstable F phenotype.

Recombinant MeV bearing the L454W/E455G F behaved similarly to WT virus, in that infection in brain organoids was limited, and in the 10- to 20-day span of two separate experiments resulted in partial negative selection of the E455G mutation. It is possible that a longer infection could result in complete elimination of the E455G mutation or introduction of additional mutations. Despite several attempts, a virus bearing purely E455G F could not be recovered, and we gather that the increased stability of the E455G F may be detrimental to fitness. The factor(s) that leads to positive selection of viruses bearing the L454W F over the L454W/E455G F in *ex vivo* models remains to be explored.

The MeV bearing an N462K F, which is far less stable than WT F and grows well in cultured cells, surprisingly, was not well suited to growth in human brain organoids. The double mutant MV L454W/G506E F seems to have reached a “balance” that makes it fit for both culture conditions. We previously described a virus from an SSPE case in which the consensus F (SSPE F) had 6 amino acid changes (G168R, E170G, S262G, A440P, R520C, L550P) (48). The SSPE F was less thermally stable than the WT F and could mediate fusion in the absence of a known receptor. When we assessed the effect of the mutations individually, we observed that no mutation alone could confer the fusion property. Some of the mutations increased the stability of the prefusion F (e.g., E170G), while others decreased it (e.g., S262G). We speculate that *in vivo* evolution leading to the fully CNS-adapted SSPE F may have occurred in the clinical case.

Deep sequencing of one of the clinical samples from the patient cohort where the L454W mutation in F was first identified revealed the presence of several other mutations in the F protein. One of these (M337L) had an allele frequency similar to that of L454W, and the F protein bearing M337L/L454W matched the fusion phenotype of the F bearing the L454W/G506 mutations, suggesting *in vivo* viral evolution toward a similar functional phenotype of F to that observed in the brain organoids. Distinct mutations that arrive at a similarly labile and receptor-independent F may be positively selected in the CNS. Mapping the M337L mutation onto the prefusion structure of MeV F (Fig. S1) reveals that this residue could form hydrophobic interactions with L256 and L257 to stabilize the prefusion conformation of MeV F.

MeV is a uniquely human pathogen, and there is no *in vivo* model that faithfully reproduces the CNS sequelae of MeV infection. The best model for acute measles infection is the nonhuman primate; however, in rhesus monkeys, cynomolgus macaques, and squirrel monkeys, no CNS manifestations have been described (21, 49–55). The murine organotypic brain culture and the human brain organoid models described here contain the four major types of CNS cells (see Fig. S3 and S6) and offer the advantage that viral spread can be monitored in real time (35). Infection in human brain organoids reveals that the single L454W mutation in MeV F is sufficient to drive human CNS infection. Infection of human brain organoids induced an innate immune response that correlated with the intensity of infection but did not prevent spread of the MeV variants with hyperfusogenic F proteins.

Infection of mouse OBC also produced an innate immune response, and in these OBC, viral titers were reduced compared to those of IFNAR1^KO^ OBC (Fig. 2L), suggesting that the innate antiviral response in the immunocompetent OBC slowed viral spread (Fig. S4A and B). The increased innate antiviral response to the L454W F MeV variant, in both human brain organoids and murine cerebellar OBC, is likely related to the higher viral titer and spread, given that most of the upregulated genes are type I interferon-inducible genes (i.e., MX1, OAS, IFIT1, etc.) (56). However, we did not have the opportunity to compare these findings to infection with other viruses, and this is an area for future investigation. An enhanced type I IFN response in viral encephalitides has been observed to correlate with the severity of CNS infection (57), and we speculate that the L454W F MeV variant may induce a brisk innate immune response by infecting a larger area of CNS tissue.

In human cases of MeV infection of the CNS, it is unclear how MeV gains access to this site. The pathway may rely on lymphocytes that cross the blood-brain barrier (BBB) (58), on infection of endothelial cells of the BBB (59, 60), or on nectin 1-positive cells from the CNS capturing membranes and cytoplasm from the surface of adjacent cells via a transendocytosis mechanism (61). To date, such mechanisms do not explain how cell-to-cell spread occurs within the CNS. Alterations in the viral fusion complex have been linked to CNS adaptation and neuropathogenesis (27). The timing of these alterations (whether they occur within the CNS or facilitate CNS infection) is also unclear. Here, we show that viruses bearing the MIBE-derived L454W F and the SSPE-derived T461I F are advantaged in infecting and spreading from cell to cell in models of CNS infection. Only the viruses bearing a dysregulated fusion complex were positively selected in the CNS.

## MATERIALS AND METHODS

### Ethical statement.

All *ex vivo* experiments with mice were performed by C.M. and M.F. (accredited by the French veterinary service) according to the French National Charter on the ethics of animals. Animals used for *ex vivo* tissues in this study were directly euthanized by decapitation according to the AAALAC recommendations and according to French Ethical Committee (CECCAPP) regulations, accreditation no. CECCAPP_ENS_2014_034. All experiments involving human cells were approved by the Columbia University Institutional Review Board (IRB) and Embryonic Stem Cell Research Oversight (ESCRO) committees.

### Peptides and chemicals.

MeV F-derived fusion inhibitory peptides were previously described (36). Briefly 36-amino acid (aa) peptides derived from the heptad repeat region at the C terminus of the MeV F protein were synthesized. Dimeric cholesterol conjugated (HRC4) forms of the peptides were used in this study. *N*-(3-cyanophenyl)-2-phenylacetamide (also known as 3G) was commercially acquired from ZereneX Molecular Limited (United Kingdom). The purity of 3G was tested by high-pressure liquid chromatography (HPLC) and shown to be >95% pure.

### Plasmids and reagents.

The genes of MeV IC323 H and F proteins were codon optimized, synthesized, and subcloned into the mammalian expression vector pCAGGS. Plasmids encoding nectin 4, CD150, were commercially acquired.

### Cells.

HEK293T (human kidney epithelial), 293-3-46 (62, 63), Vero, and Vero-SLAM/CD150 (African green monkey kidney) cells were grown in Dulbecco’s modified Eagle’s medium (DMEM; Life Technologies, Thermo Fisher Scientific) supplemented with 10% fetal bovine serum (FBS; Life Technologies, Thermo Fisher Scientific) and antibiotics at 37°C in 5% CO_2_. The 293-3-46 and Vero-SLAM/CD150 culture media were supplemented with 1 mg/ml Geneticin (Thermo Fisher Scientific).

### Recombinant virus production and analysis.

MeV IC323-EGFP (64) is a recombinant MeV expressing the gene encoding EGFP. All variants with the mutations T461I, N462K, L454W, and L454W/E455G were generated in the MeV IC323-EGFP background using reverse genetics (using the plasmid encoding MeV IC323-EGFP, kindly provided by Yanagi, Kyushu University, Fukyoka, Japan). MeV IC323-TdTomato was generated by replacing the EGFP expression cassette with the sequence coding for tdTomato red fluorescent protein. MeV IC323 recombinant viruses were rescued in 293-3-46 cells as previously described (63). Production of the virus bearing the L454W was performed at either 37°C or 32°C. All viruses were propagated and titrated in Vero-SLAM/CD150 cells.

### Structural modeling.

Twenty models were produced for the wild-type (WT) measles virus fusion glycoprotein (MeV F) using the protein homology server Phyre2 (65). Bad local geometries of each model for prefusion and postfusion states of MeV F were manually fixed using the program XtalView (66). The resulting models were subsequently refined using CNS-1.3 (67) against the electron density of 5EVM for the prefusion state. The same methodology was used for the production of models for all MeV-F mutant proteins. All structural figures were produced using PyMol (http://www.pymol.org/).

### Beta-Galactosidase (Beta-Gal) complementation-based fusion assay.

The Beta-Gal complementation-based fusion assay was performed as previously described (15). Briefly, HEK293T cells transiently transfected with the constructs indicated above and the omega reporter subunit were incubated for the indicated period with cells coexpressing viral glycoproteins and the alpha reporter subunit (36).

### Cell surface staining with F-conformation-specific MAbs.

HEK293T cells transiently transfected with viral glycoprotein constructs were incubated overnight at 37°C in complete medium (DMEM, 10% FBS). Then, 20 h posttransfection, cells were transferred to 55°C for the times indicated in the figures. Thereafter, cells were incubated with mouse monoclonal antibodies (MAbs) that specifically detect MeV F in its prefusion conformation (1:1,000) (44, 68, 69) for 1 h on ice. Cells were washed with phosphate-buffered saline (PBS) and then incubated for 1 h on ice with Alexa-488 anti-mouse secondary antibody (1:500; Life Technologies). Cells were washed with PBS and then fixed for 10 min on ice with 4% paraformaldehyde (PFA) with a 1:1,000 dilution of DAPI (4′,6-diamidino-2-phenylindole; Thermo Fisher) for 60 min. Plates were washed, 0.01% sodium azide was added, and plates were imaged via the use of an IN Cell Analyzer. The percentages of positively recognized cells were determined using Cell Profiler.

### Organotypic cerebellar culture preparation and treatment postinfection.

Cerebellar slices were prepared from IFNAR1^KO^ (and SLAM/CD150tg-IFNAR1^KO^) or C57BL/6 mice and maintained in culture as detailed elsewhere (33). Briefly, cerebella were isolated from the brains of 7-day-old mice and cut with a McIlwain tissue chopper (WPI-Europe) to obtain 350-μm-thick progressive slices. The brain slices were then dissociated in cold Hibernate-A/5 g/liter d-glucose/1× kynurenic acid buffer and laid out on Millipore cell culture insert membranes (Millicell cell culture insert, 30 mm, hydrophilic polytetrafluoroethylene). Slices were subsequently cultured in GlutaMAX minimal essential medium supplemented with 25% horse serum, 5 g/liter glucose, 1% HEPES (all Thermo Fisher Scientific), and 0.1 mg/liter human recombinant insulin (R&D Systems) at 37°C in 5% CO_2_ in a humidified atmosphere. The medium was changed every day after the slicing procedure. Slices from 5 mice were infected on the day of slicing with the indicated recombinant viruses (5.10^3^ PFU/slice from IFNAR1^KO^ and 100 PFU/slice from SLAM/CD150tg x IFNAR1^KO^ mice). Cultures were then treated daily from day 1 to day 4 either with serial dilutions of HRC4 fusion inhibitor in neurobasal medium or with vehicle (untreated condition; “NT”). Then, 2 μl of 10,000 nM, 1,000 nM, or 100 nM HRC4 was added on top of each of the 5 slices in each well. After several minutes, the drops containing the peptides were completely absorbed and reached the lower compartment of the system that contains the feeding medium. The final concentration in the culture medium (1 ml) was 100 nM, 10 nM, or 1 nM. At each time point, slices were collected, RNA extracted, and RT-qPCR performed as previously described (34).

### Human brain organoid differentiation.

Human brain organoids were generated as previously described (38) from two iPSC lines (FA0000010 and FA0000011, for brevity called FA10 and FA11). Briefly, cells were dissociated into single cell suspension with Accutase (Stem Cell Technologies; catalog [cat.] no. 7920) and seeded at 4,500 cells/well in 96-well low-attachment U-bottom plates, using embryoid body (EB) medium (DMEM/F12; Thermo Fisher Scientific; cat. no. 11330-032) supplemented with 20% knockout serum replacement (Thermo Fisher Scientific; cat. no. 10828-028), 3% embryonic stem cell (ES)-quality batch-tested fetal bovine serum (Thermo Fisher Scientific; cat. no. 10439024), 1% Glutamax (Thermo Fisher Scientific; cat. no. 35050-038), 1× nonessential amino acids, 0.1 mM 2-mercaptoethanol, 4 ng/ml basic fibroblast growth factor (bFGF; R&D Systems; cat. no. 233FB01M), and 50 μM Y-27632. Fresh medium was replaced every other day until day 6. On day 6, EB medium was replaced with neural induction (NI) medium (DMEM/F12, 1× N2 supplement, 1× Glutamax, 1× nonessential amino acid [NEAA], and 1 μg/ml heparin [Sigma-Aldrich; cat. no. H3149]), and the organoids were transferred to 60- mm or 100-mm low-attachment plates. The organoids were allowed to form neuroepithelium tissue until day 11 to 14, with medium changes every other day. Between day 11 and 24, the organoids were coated with Matrigel droplets and allowed to gel by keeping them at 37°C for 30 min. Matrigel-coated organoids were transferred to differentiation medium (1:1 DMEM/F12:neurobasal, 0.5% N2 supplement, 2% B27 supplement without vitamin A [Life Technologies; cat. no. 12587010], 0.25% insulin solution [Life Technologies; cat. no. 12585014], 50 μM 2-mercaptoethanol, 1% Glutamax, 0.5% NEAA, 1% penicillin-streptomycin), for 4 days. After 4 days, organoids were transferred to differentiation medium containing B27 supplement with vitamin A (Thermo Fisher Scientific; cat. no. 17504-044). Brain organoids were cultured for an additional 60 days with medium changes every 7 days and used for experiments at 90 days.

### Immunofluorescent staining.

Murine organotypic brain cultures (OBC) from 7-day-old SLAM mice were fixed for 1 hour in 4% paraformaldehyde (PFA), washed in 1× Dulbecco’s phosphate-buffered saline (DPBS), dehydrated, clarified in xylene, and embedded in paraffin. Slices 10 μm thick were dewaxed thrice for 3 min in xylene and rehydrated following this protocol: ethanol 100% twice for 3 min, ethanol 95% for 3 min, ethanol 70% for 3 min, ethanol 30% for 3 min, and PBS 1× (twice for 3 min). Antigen unmasking was performed in sodium citrate buffer (at 100°C for 20 min) and slowly cooled down to room temperature (RT). The slices were permeabilized and blocked in 1× DPBS-3% BSA-0.3% Triton X-100 (permeabilization and block solution) for 1 h at RT. Slices were incubated in the permeabilization and block solution containing the primary antibodies overnight at 4°C. After 3 washes (for 5 min each) in 1× DPBS, slices were incubated in the permeabilization and block solution containing the secondary antibodies (1:750) and the DAPI (1:1,000) for 1 h at RT. After 3 washes in 1× DPBS, slices were mounted with Fluoromount-G aqueous mounting medium (SouthernBiotech; catalog no. 0100-01). Images were taken with the microscope set at epifluorescence (Eclipse Ts2R Nikon) and analyzed using ImageJ software.

Human brain organoids were picked from culture and embedded in optimum cutting temperature compound (OCT; Tissue Tek) and then cryosectioned (5 to 8 μm thickness). Brain organoid sections were thawed at room temperature for 5 min, fixed in 4% PFA for 10 min and permeabilized with 0.25% Triton X-100 in PBS for 20 min. Subsequently, the slides were blocked with 10% donkey serum in PBS for at least 1 h. All the steps were performed at RT unless stated otherwise. Sections were incubated overnight at 4°C with primary antibodies. After 3 washes for 5 min each on gentle rocking, in 0.025% Triton X-100 in PBS, sections were stained with fluorescently conjugated secondary antibodies for 1 h. Cell nuclei were stained with DAPI (1:1,000; Sigma-Aldrich), and the sections were mounted with Vectashield mounting medium (Vector Laboratories, Inc., Burlingame, CA), covered, and imaged with DMi8 (Leica Microsystems, Buffalo Grove, IL).

### Antibodies used for immunofluorescent staining.

See Table 2 for a list of antibodies.

**TABLE 2:** Antibodies used for immunofluorescent staining

Antibody and manufacturer	Source
Murine organotypic brain cultures (OBC)	
Rabbit polyclonal anti-NeuN; EMD Millipore	Cat. no. ABN78
Rabbit polyclonal anti-calbindin D-28K (CB28K); Swant	Cat. no. CB38
Mouse monoclonal anti-myelin basic protein (MBP101); Abcam	Cat. no. ab62631
Rabbit polyclonal anti-iba1; Wako	Cat. no. 016-20001
Rabbit polyclonal anti-glial fibrillary acidic protein (GFAP); Agilent Technologies, Dakonumber	Cat. no. Z0334
Goat polyclonal anti-Olig2; R&D system	Cat. no. AF2418
Alexa Fluor 555 donkey anti-mouse IgG (H + L); Thermo Fisher Scientific	Cat. no. A31570
Alexa Fluor 555 donkey anti-rabbit IgG (H + L); Thermo Fisher Scientific	Cat. no. A31572
Alexa Fluor 555 donkey anti-goat IgG (H + L); Thermo Fisher Scientific	Cat. no. A21432
4-,6/diamidino/2/phenylindole, DAPI; Thermo Fisher Scientific	Cat. no. 62248
Human brain organoids	
Rabbit anti-NeuN; Abcam	No. ab177487
Rabbit anti-Olig1; Abcam	No. ab68105
Rabbit anti-nestin; Abcam	No. ab105389
Rabbit anti- anti-glial fibrillary acidic protein (GFAP); Cell Signaling	No. 12389
Rabbit polyclonal anti-Olig2; Proteintech	No. 13999-1-AP
Chicken anti-microtubule-associated protein 2 (MAP2); Abcam	No. ab5392
Rabbit anti-Pax6; Biolegend	No. 901301
Alexa Fluor 647 donkey anti-mouse IgG (H + L); Invitrogen	No. A31573
Alexa Fluor 594 goat anti-chicken IgG (H + L); Invitrogen	No. A11042
2-(4-amidinophenyl)-6-indolecarbamidine dihydrochloride (DAPI); Sigma-Aldrich	No. D9542

### Tiled images.

For Fig. 2B to E, H and I and Fig. 4D and G, the fluorescence signal emitted was acquired from several different fields. Then, the images were analyzed with the Stitching plugin for ImageJ (70), which reconstructs images/stacks from an arbitrary number of tiled input images/stacks, yielding the best overlap in terms of the cross-correlation measure. In Fig. S7, the individual pictures used for Fig. 2I and Fig. 4G are presented.

10.1128/mBio.00799-21.7 FIG S7 Raw pictures used to reconstitute the images from Fig. 2I and 4G. (A and B) Unprocessed pictures from Fig. 2I. *Ex vivo* organotypic brain cultures (OBC) from C57BL/6 murine brains infected with MeV-IC323-L454W F EGFP (1000 PFU/slice). (A) Infection with F bearing L454W and L454W/E455G F viral quasispecies. (B) Infection with F bearing L454W and L454W/E455G viral quasispecies. Pictures were taken 7 days postinfection using a Nikon Eclipse Ts2R microscope. (C) Four pictures were acquired from each well (from a 96-well plate for the experiment in Fig. 4G) and combined in panel D. Download FIG S7, JPG file, 0.3 MB. Copyright © 2021 Mathieu et al. 2021 Mathieu et al. https://creativecommons.org/licenses/by/4.0/ This content is distributed under the terms of the Creative Commons Attribution 4.0 International license.

### Brain organoid RNA-Seq and analysis.

RNA from uninfected and infected brain organoids was extracted using Direct-zol RNA MicroPrep (Zymo) and submitted to the JP Sulzberger Columbia Genome Center for library preparation and sequencing. Strand-specific RNA-Seq libraries were prepared using a poly-A enrichment and were sequenced on an Illumina NovaSeq instrument with paired-end 2 × 100-bp reads. After quality and adapter trimming, transcript abundance quantification was performed using Kallisto v0.44.0 (71) with GRCh38 as the reference genome.

To understand the brain organoid developmental stage, we used the BrainSpan data set (79). Since BrainSpan offers normalized reads per kilobase per million (RPKM) expression values based on Gencode v10 annotations, we mapped R1 sequencing reads to GRCh37 annotation of the human genome using Bowtie2 (72) and quantified gene-level RPKM levels using featureCounts (73). Genes were filtered based on an arbitrary gene-level RPKM sum of greater than 1,000 across all 539 RNA-Seq experiments (524 BrainSpan, 15 MeV infections). All-by-all correlation matrices of log_2_-transformed RPKM values with a pseudocount of 1 were generated in R v3.6.2. The top 100 BrainSpan samples with the highest correlation coefficients with uninfected FA10 brain organoid replicate 1 were pulled, and a heatmap of correlation coefficients was generated using pheatmap (https://github.com/raivokolde/pheatmap).

Differential gene expression analysis was performed using the Kallisto transcript abundances and the R Bioconductor package DESeq2 (74). The code for analysis is available at http://www.github.com/greninger-lab/MeV-brain-organoids. Low-count filtering was performed on genes with an average of less than one count per sample. Batch effects and biological differences between organoids were incorporated into the design formula as confounders, and normalization and differential expression analysis was performed using DESeq2 with default parameters. The expression of the 50 genes with the lowest Benjamini-Hochberg adjusted *P* value between MeV IC323-EGFP-F L454W and uninfected organoids was plotted on a heatmap generated using the R package pheatmap, with bar graphs generated in the R package ggplot. Enrichment of differentially expressed genes (padj, <0.0001, absolute value log_2_ fold change, >1) between MeV IC323-EGFP-F L454W and those uninfected in KEGG pathways was evaluated using the R package ReactomePA (75). To calculate the reads per million (RPM) values for MeV reads, each sample was aligned against the NC_001498 MeV reference sequence using Bowtie2 with default parameters (72). MeV RPM values were calculated using the number of mapped reads in the resulting BAM file.

### RNAseq of mouse brain slices.

RNA extracted from brain slices was prepared and sequenced as for organoids, as described above. Reads were pseudoaligned with Kallisto v0.44 to mouse reference transcriptome mmGRCm38. Differential expression analysis was performed in DESeq2, incorporating batch effects into the design formula. An expression heatmap was generated as for brain organoids, described above. MeV RPMs were calculated as described above.

### Brain organoid genome-specific RT-qPCR.

Specific reverse transcription targeting the MeV genomic strand was performed with the SuperScript III first-strand synthesis system (Thermo Fisher), according to the manufacturer’s instructions, on 500 ng of total RNA using reverse primer for human GAPDH and forward (FW) 5′ tagged-MeV primer 5′-gcagggcaatctcacaatcaggAAAACTGGTGTTCTACAACAA-3′ containing MeV sequence of the antigenomic strand with a TAG sequence from Nipah virus. The obtained cDNAs were then diluted 1:10. qPCRs were then performed as described previously (76) using MeV reverse (Rev) 5′-TGAAGGCCACTGCATT-3′ and Tag FW 5′-gcagggcaatctcacaatcagg-3′ primers. All results were normalized to human GAPDH deviation.

### Metagenomic Next-Generation Sequencing (mNGS) and variant calling.

The mNGS was performed as previously described (76). Briefly, RNA was extracted from 50 μl of viral culture using the Quick-RNA viral kit (Zymo) and treated with TURBO DNase I (Thermo Fisher). cDNA was generated from the DNase-treated RNA using Superscript IV reverse transcriptase (Thermo Fisher) and random hexamers (IDT), followed by second-strand synthesis via Sequenase v2.0 DNA polymerase. The resulting double-stranded cDNA was then purified with the DNA Clean & Concentrator kit (Zymo). Libraries were constructed from 2 μl of cDNA using the Nextera XT kit (Illumina) and sequenced on 1 × 192-bp Illumina MiSeq runs.

Sequencing reads were adapter and quality trimmed using Trimmomatic v0.38 (77). Variants present at a frequency greater than 10% and coverage greater than 10× were identified with LAVA (https://github.com/greninger-lab/lava) using the MeV reference genome (GenBank accession no. NC_001498). All variants were manually confirmed by mapping sequencing reads to the same MeV reference strain in Geneious v11.1.4 (78). Those variants present in intergenic region between the matrix and fusion proteins as well as in homopolymeric tracts were excluded from the analysis. Sequencing reads are available under NCBI BioProject number PRJNA594952.

### Statistical analysis.

All other statistical comparisons were performed using the Mann-Whitney U-test. All analyses were performed in GraphPad Prism v5 software. Statistical analysis of RNAseq data was performed in R.

10.1128/mBio.00799-21.8 DATA SET S1. Murine OBC LAVA plots. Longitudinal sequence analysis of measles viruses in murine OBC. Download Data Set S1, ZIP file, 0.4 MB. Copyright © 2021 Mathieu et al. 2021 Mathieu et al. https://creativecommons.org/licenses/by/4.0/ This content is distributed under the terms of the Creative Commons Attribution 4.0 International license.

10.1128/mBio.00799-21.9 DATA SET S2. Human brain organoid LAVA plots. Longitudinal sequence analysis of measles viruses in human brain organoids. Download Data Set S2, ZIP file, 1.3 MB. Copyright © 2021 Mathieu et al. 2021 Mathieu et al. https://creativecommons.org/licenses/by/4.0/ This content is distributed under the terms of the Creative Commons Attribution 4.0 International license.
