# Supplementary figures and images for: Molecular Features of the Measles Virus Viral Fusion Complex That Favor Infection and Spread in the Brain
Source: mBio. 2021 Jun 1;12(3):e00799-21. doi: 10.1128/mBio.00799-21 (PMC8263006; doi:10.1128/mBio.00799-21)

**Pre-fusion MeV F**

**Model of Observed MeV F Mutations**

**A**

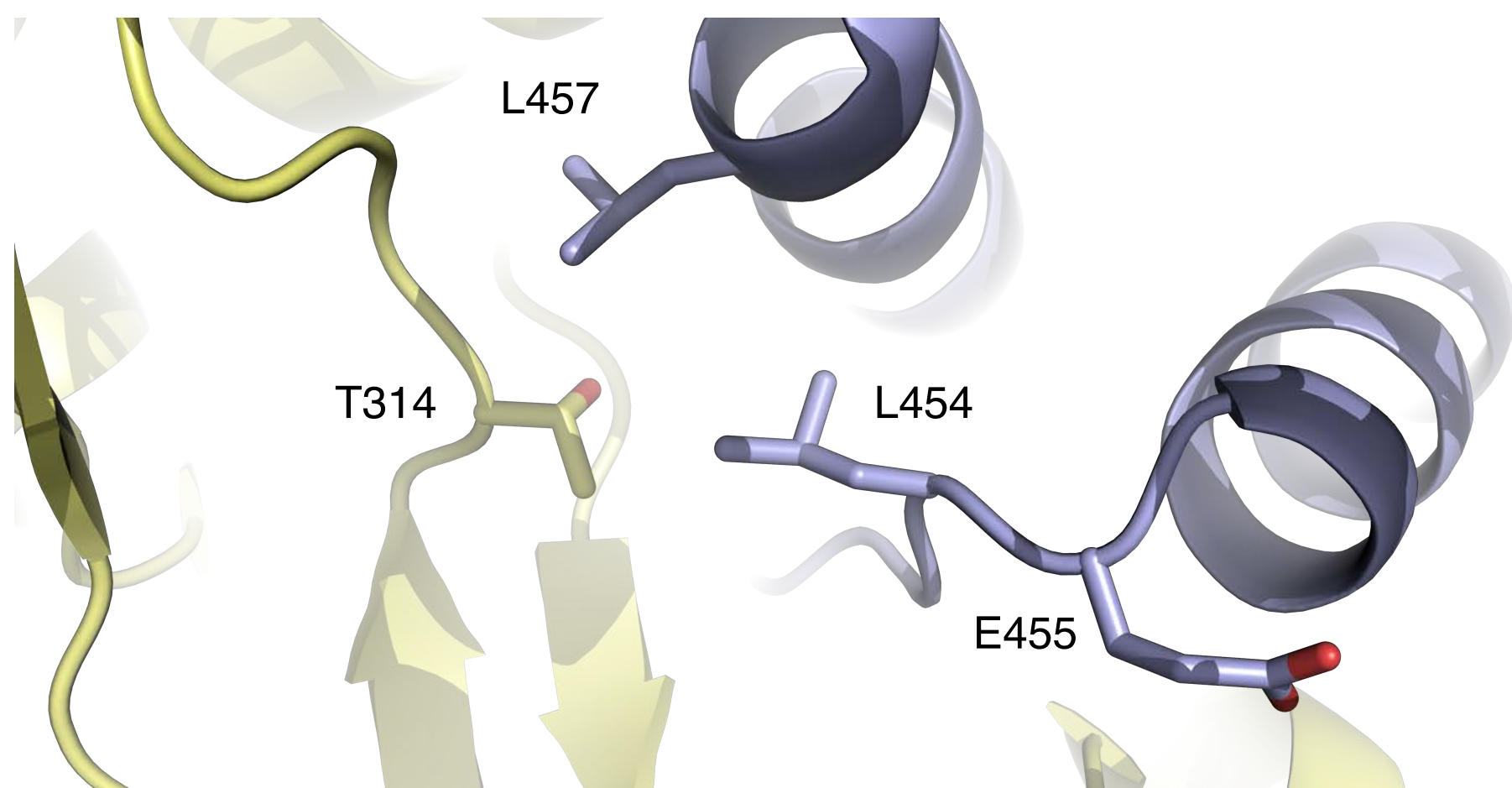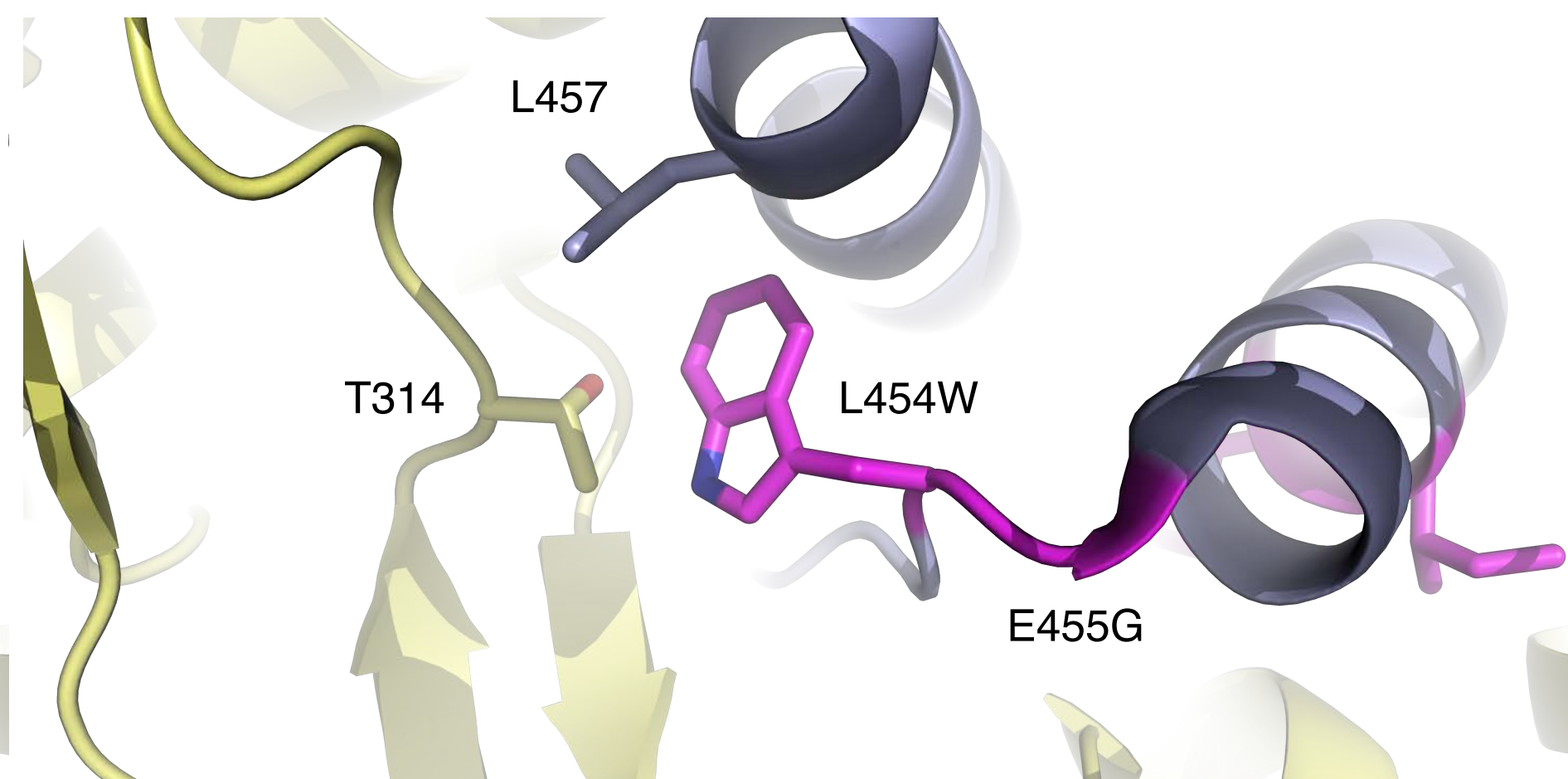

**B**

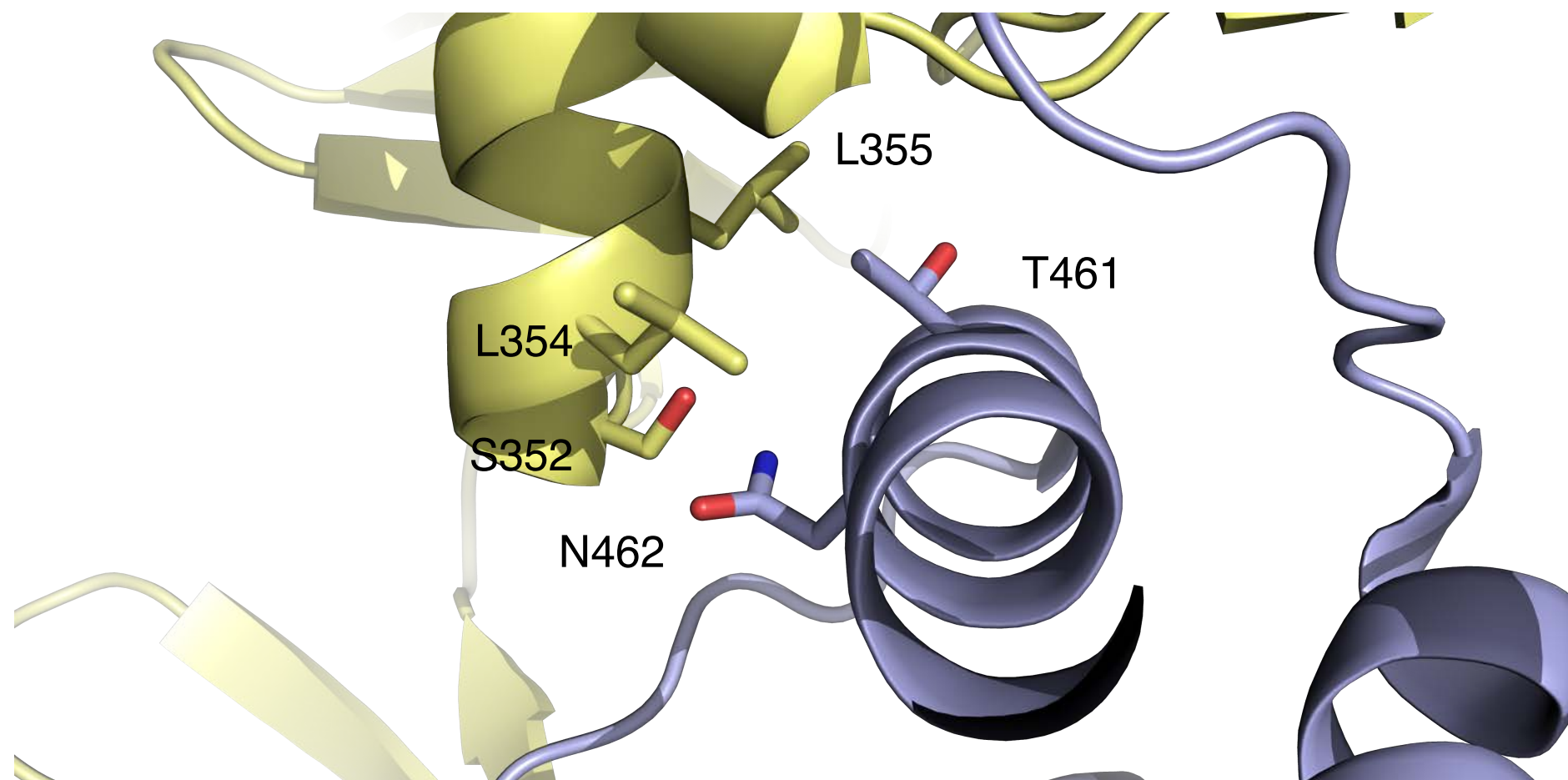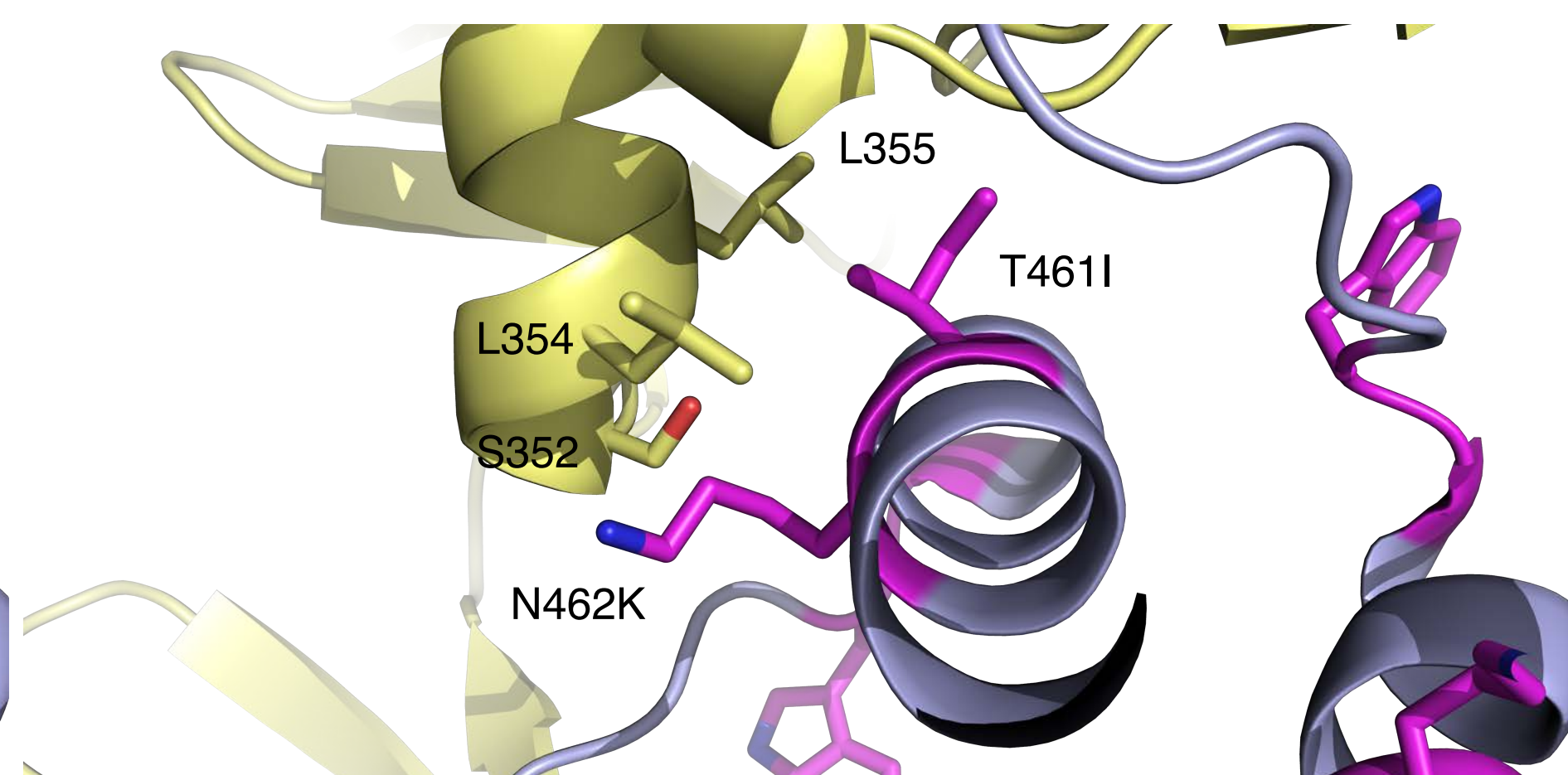

**C**

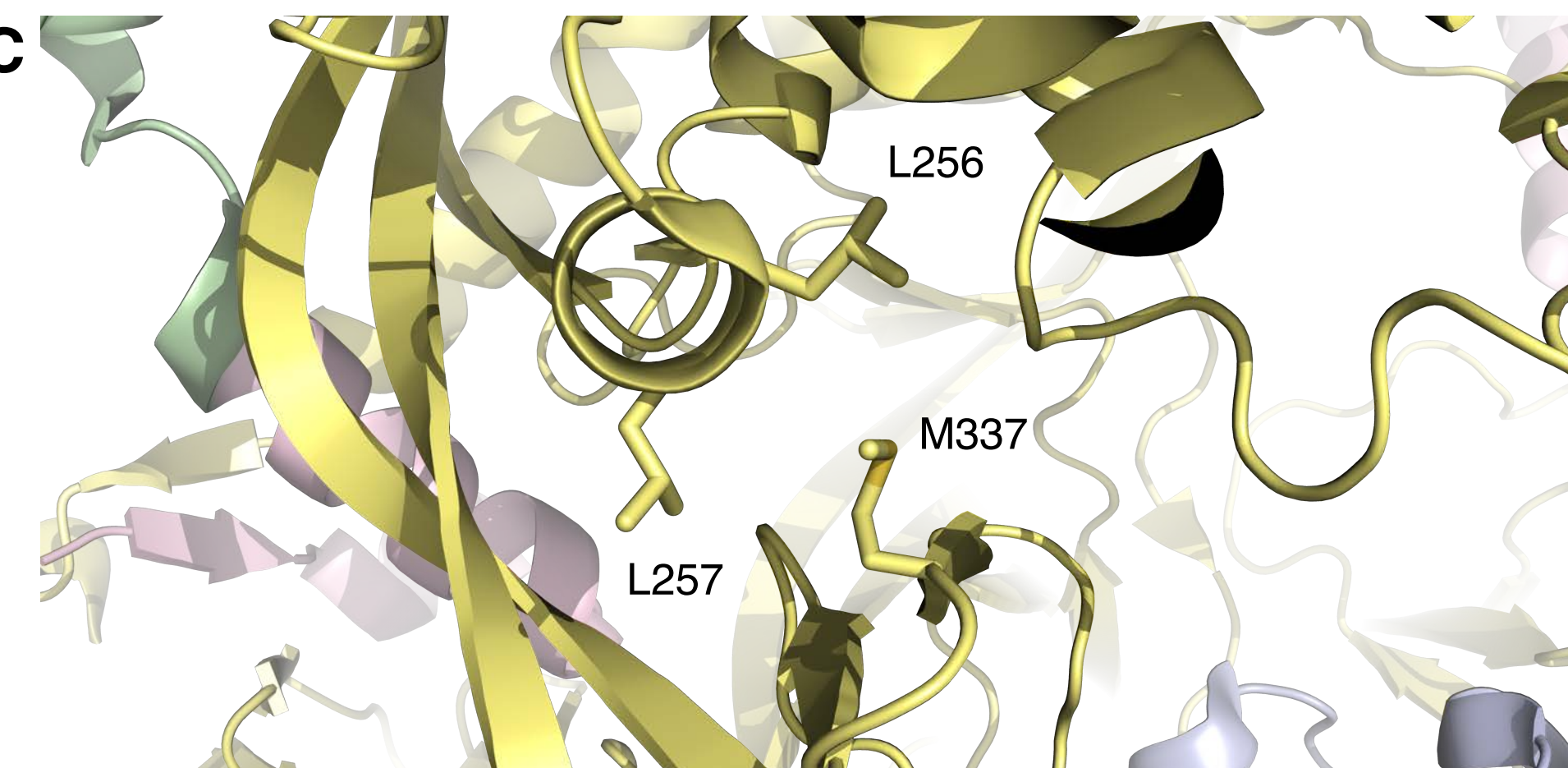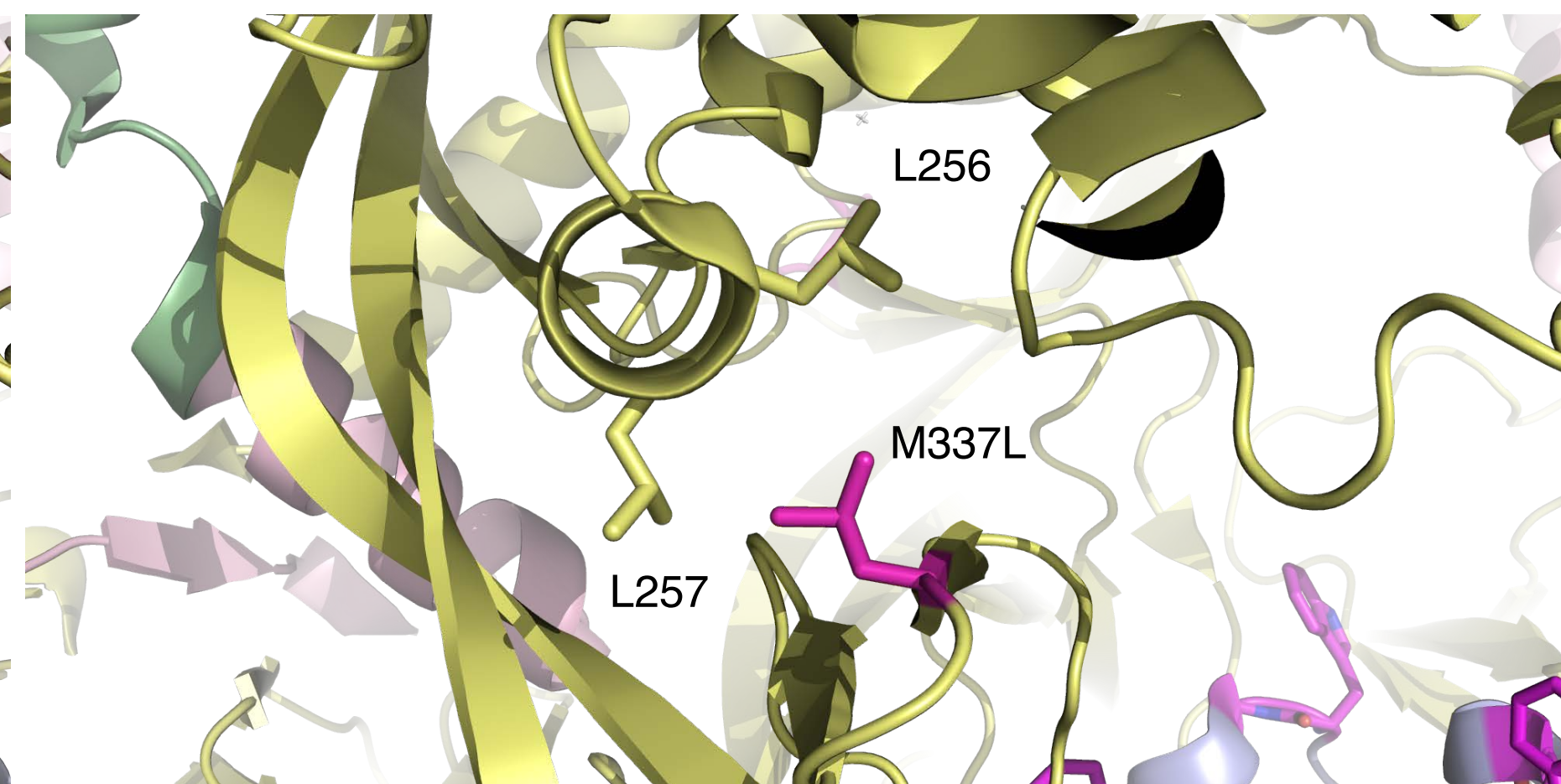

Supplement: FIG S1 [file mbio.00799-21-sf001.pdf]

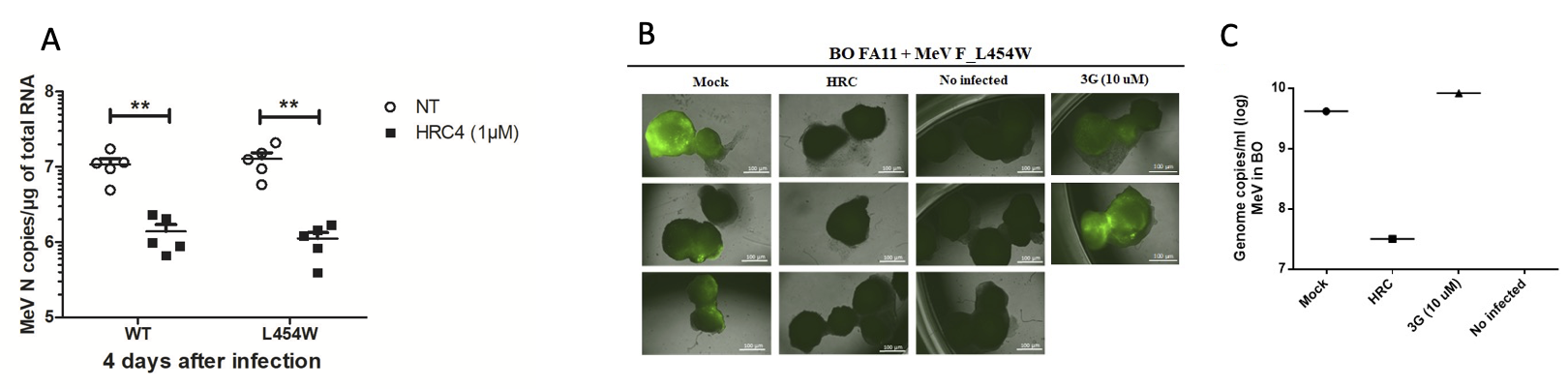

Supplement: FIG S2 [file mbio.00799-21-sf002.tif]

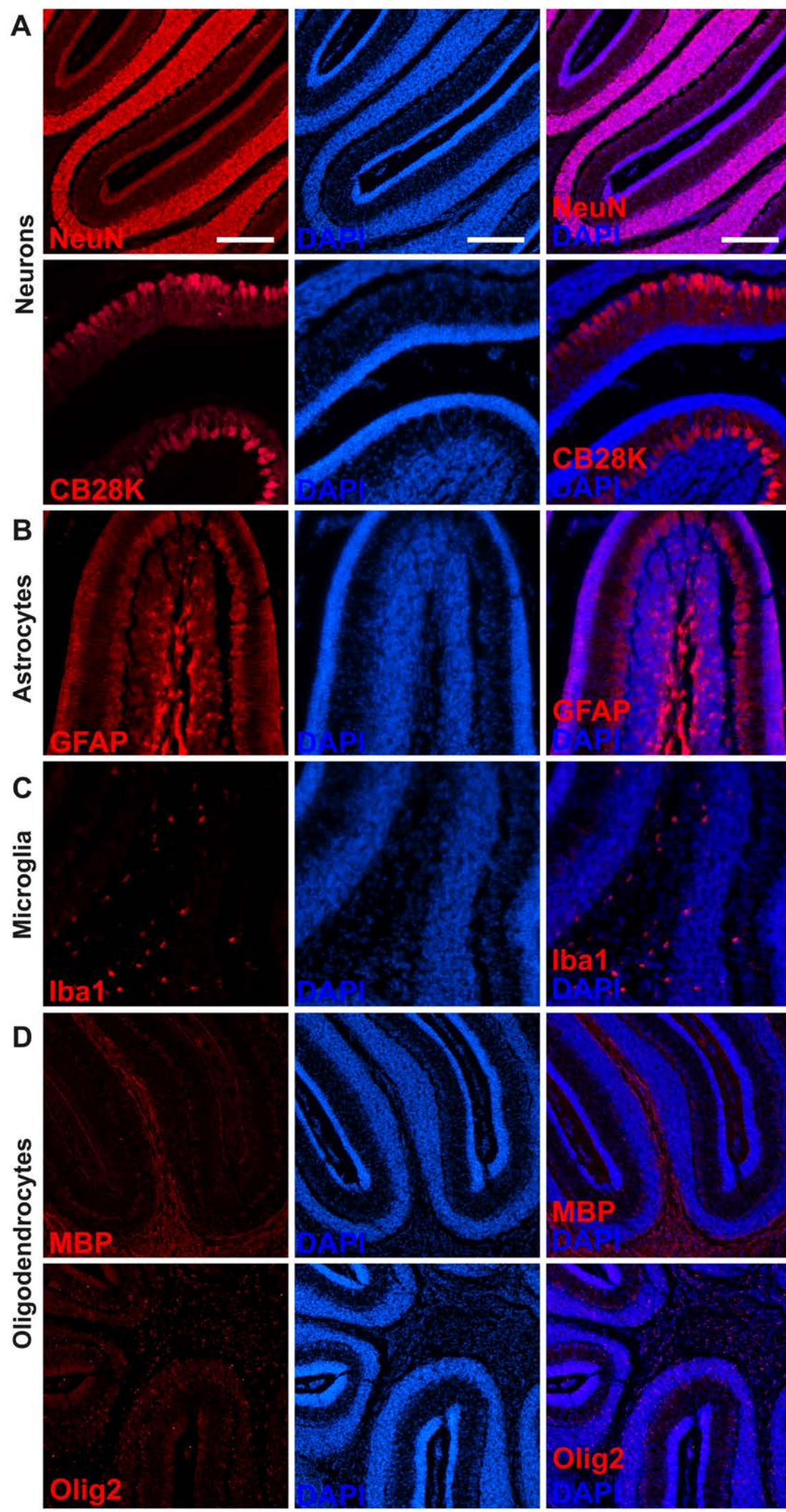

Supplement: FIG S3 [file mbio.00799-21-sf003.pdf]

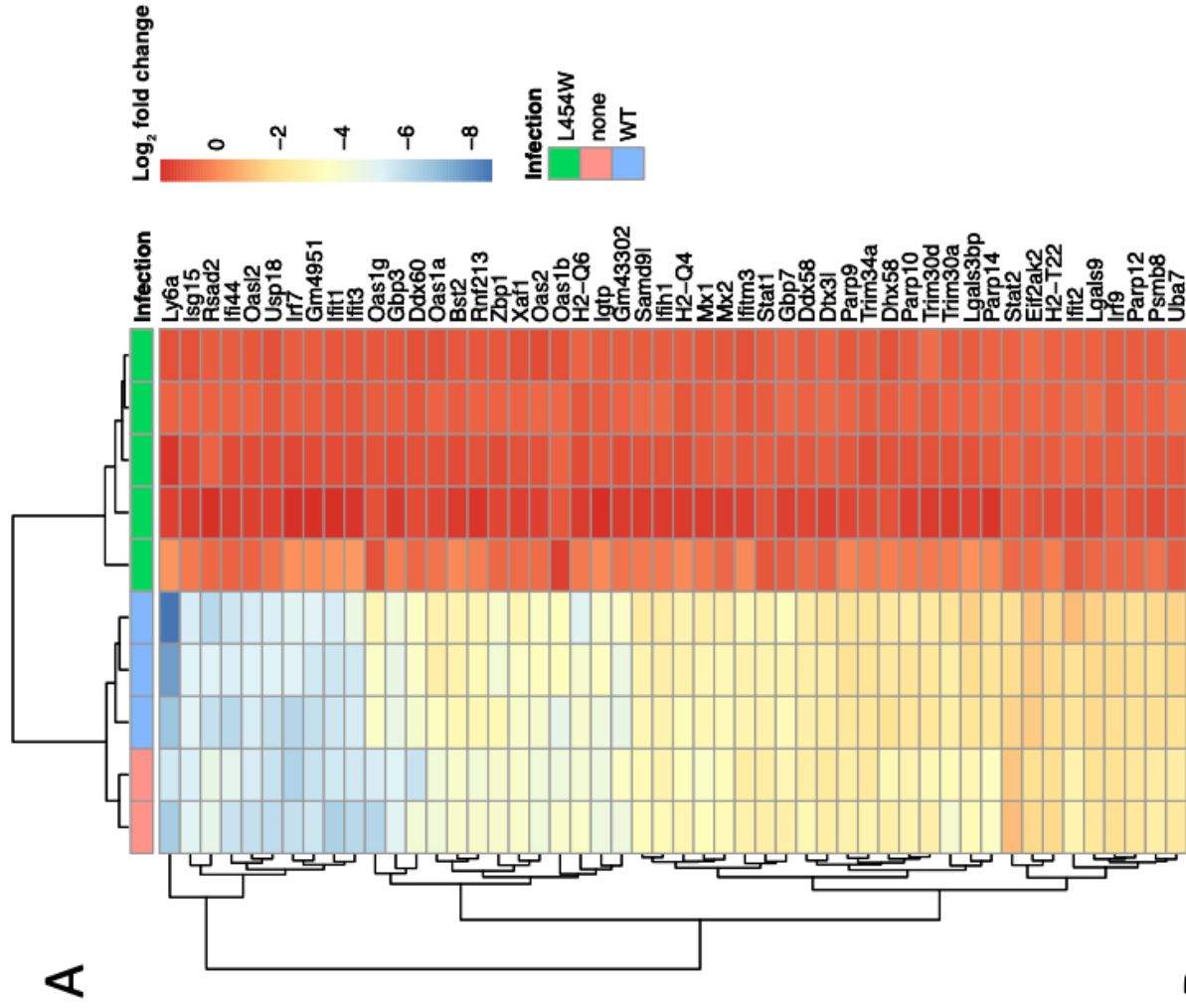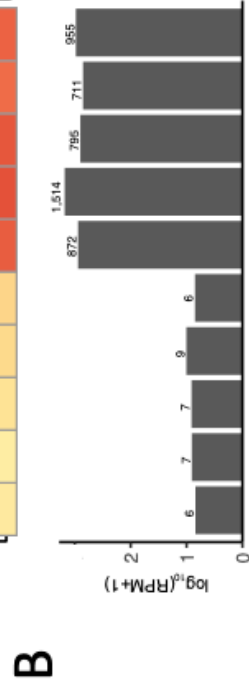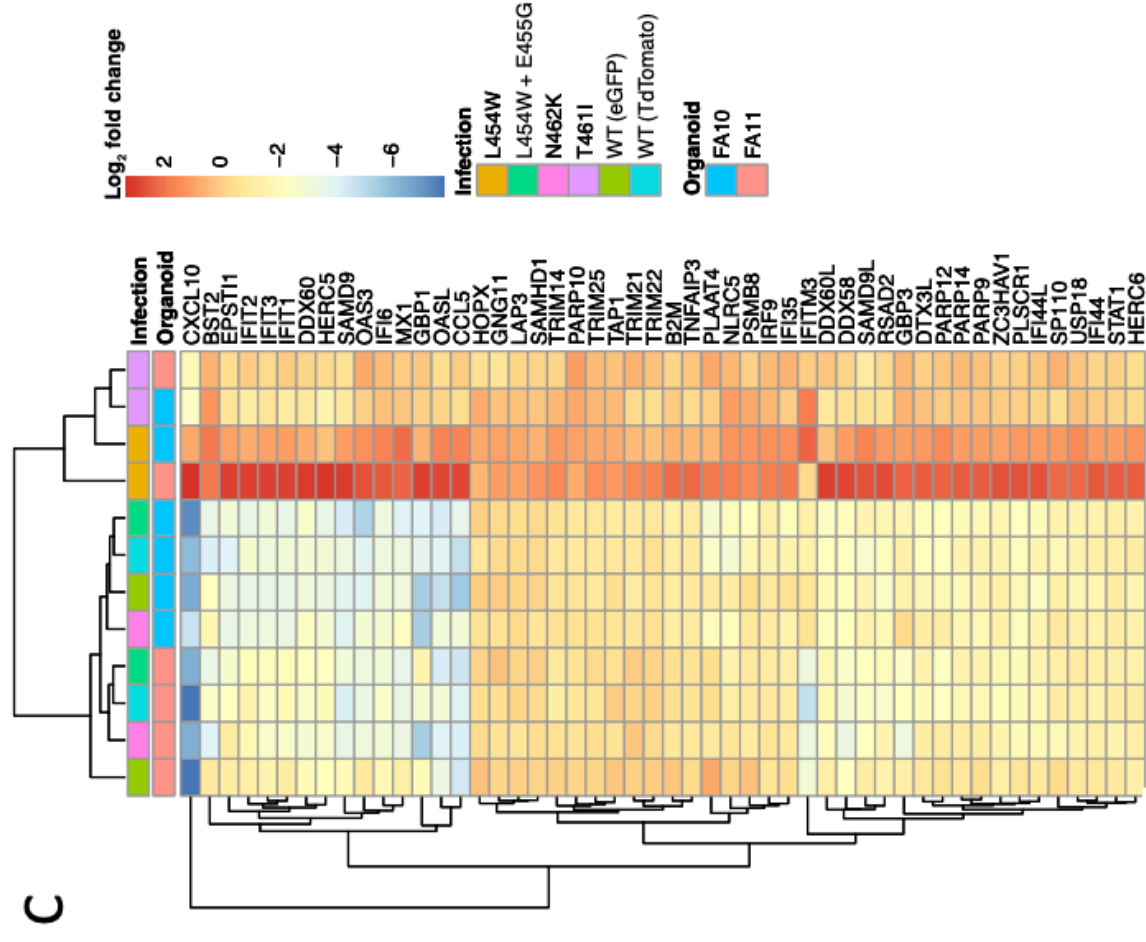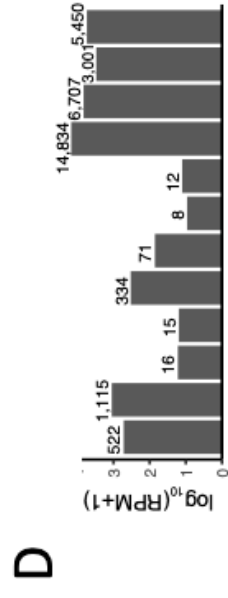

Supplement: FIG S4 [file mbio.00799-21-sf004.pdf]

A

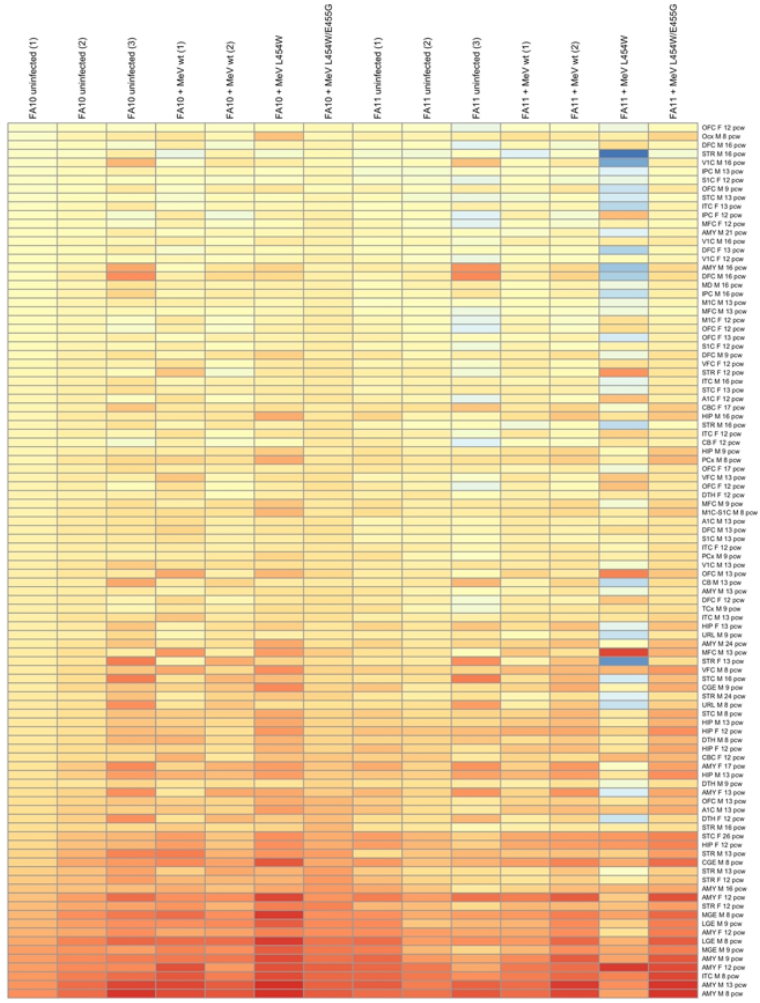

B

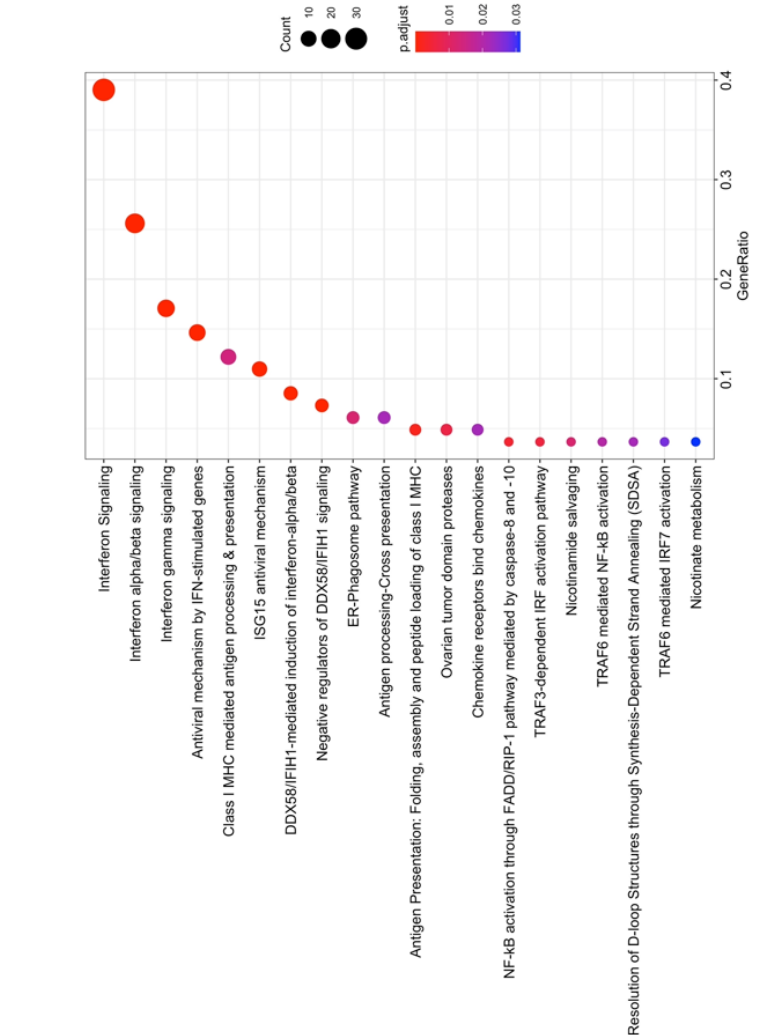

Supplement: FIG S5 [file mbio.00799-21-sf005.pdf]

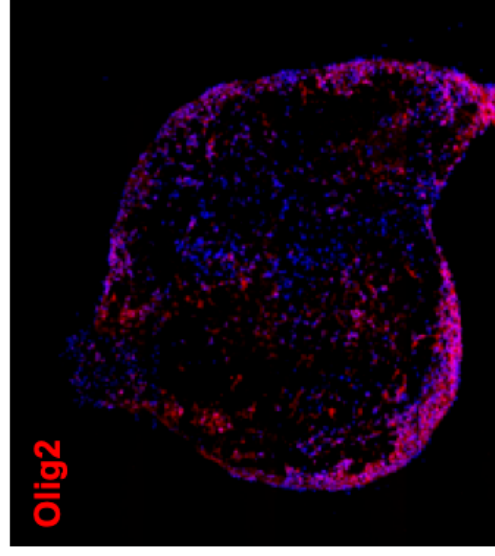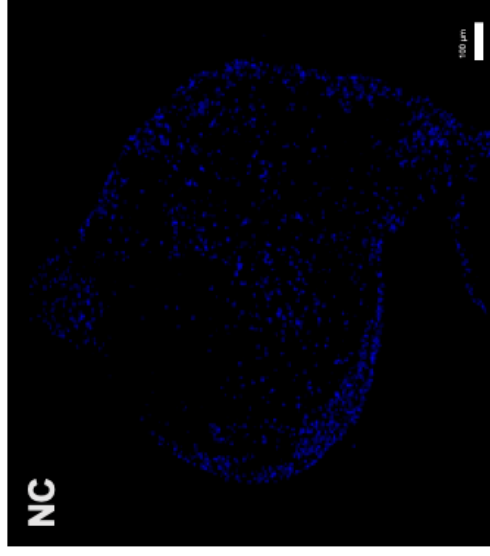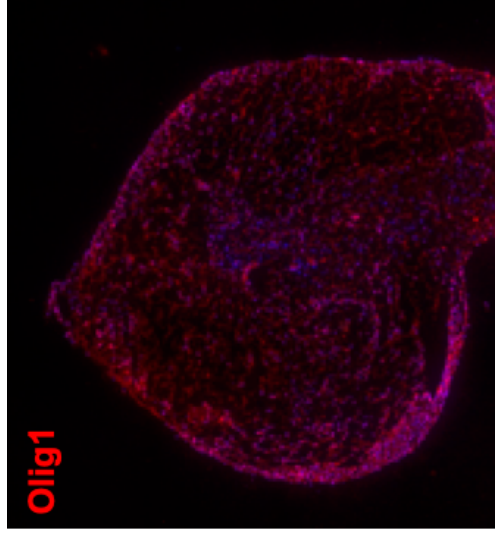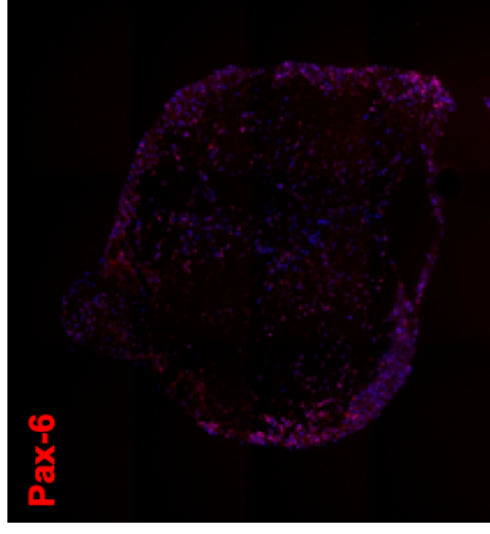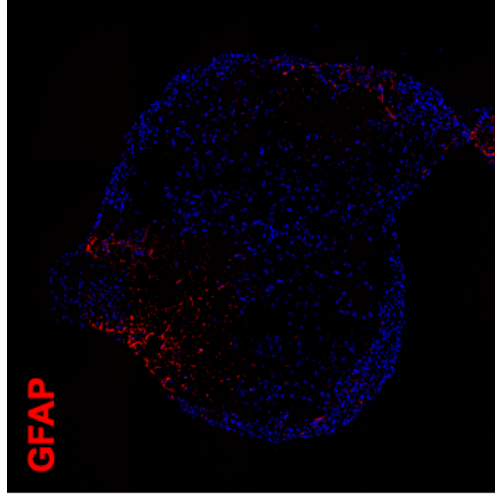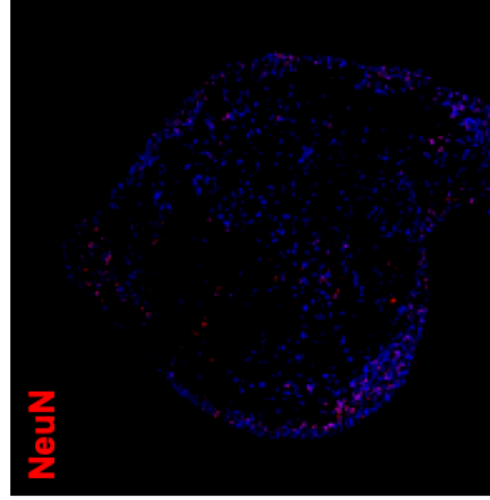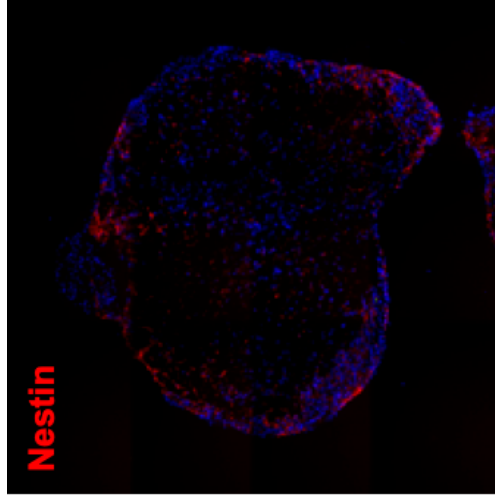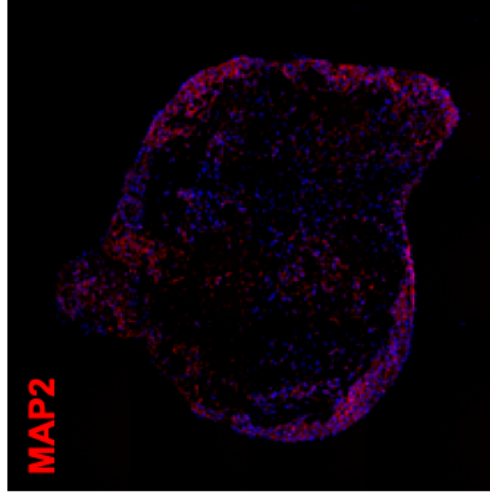

Supplement: FIG S6 [file mbio.00799-21-sf006.pdf]

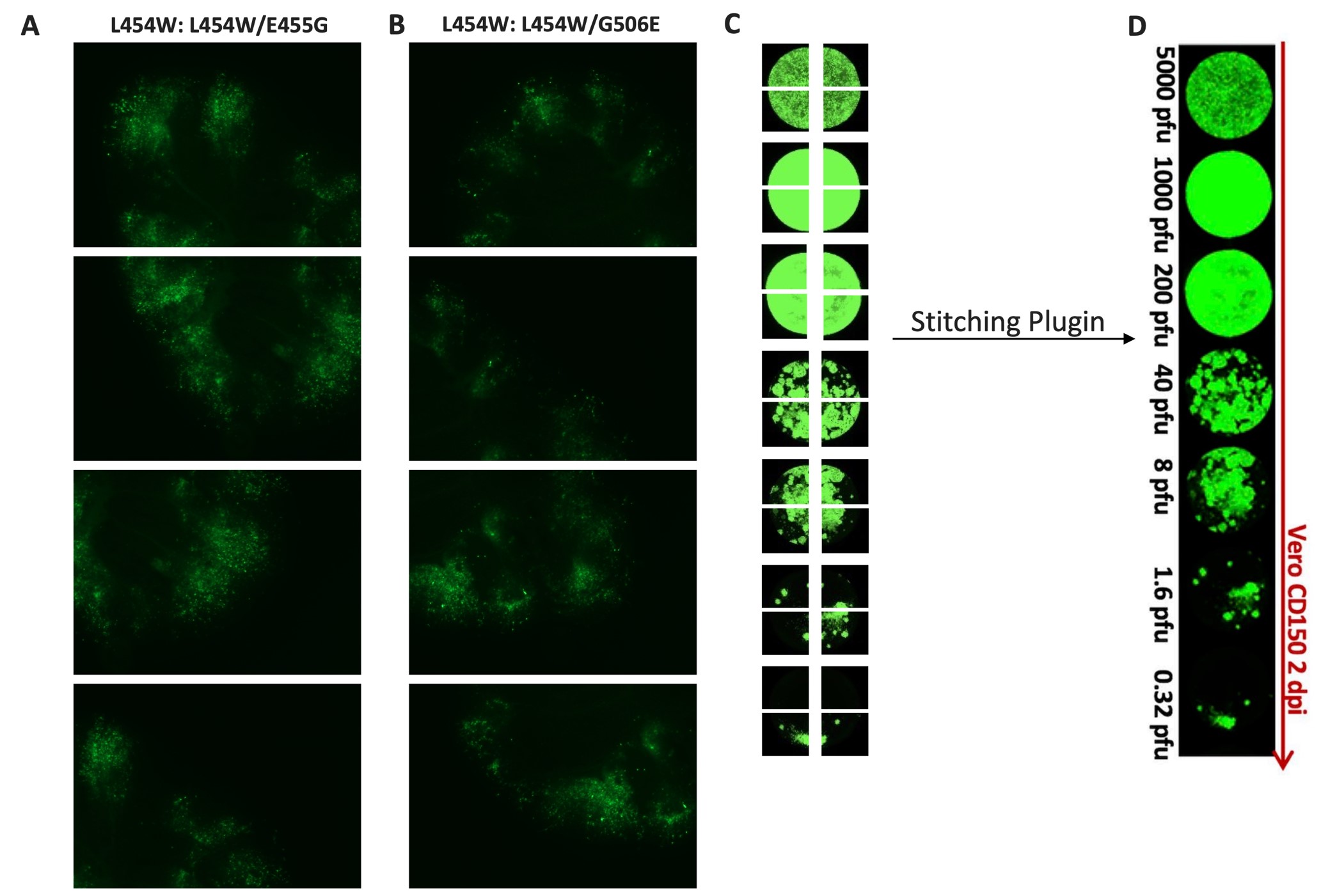

Supplement: FIG S7 [file mbio.00799-21-sf007.jpg]
