# Supplementary material for: Molecular Features of the Measles Virus Viral Fusion Complex That Favor Infection and Spread in the Brain
Source: mBio. 2021 Jun 1;12(3):e00799-21. doi: 10.1128/mBio.00799-21 (PMC8263006; doi:10.1128/mBio.00799-21)
Supplement: DATA SET S2. [file mbio.00799-21-sd002.zip › data s2.html]

Brain Organoids Combined LAVA
